# Hantavirus is Associated With Open Developed Areas and Arid Climates, Highlighting Increased Risk in the Western United States

**DOI:** 10.1155/tbed/7126411

**Published:** 2025-10-16

**Authors:** Morgan E. Gorris, Amy Whitesell, Carson Telford, Trevor Shoemaker, Andrew W. Bartlow

**Affiliations:** ^1^Information Systems and Modeling, Los Alamos National Laboratory, Los Alamos, New Mexico, USA; ^2^Viral Special Pathogens Branch, U.S. Centers for Disease Control and Prevention, Atlanta, Georgia, USA; ^3^Genomics and Bioanalytics, Los Alamos National Laboratory, Los Alamos, New Mexico, USA

## Abstract

In the United States, hantaviruses can cause hantavirus pulmonary syndrome (HPS) in humans, an acute respiratory illness with a high mortality rate. Most people contract HPS from exposure to infected rodent excrement. The interannual dynamics of hantavirus transmission are tied to both environmental and human-related factors, including changes in annual climate conditions, rodent populations, and the built environment in which humans are more likely to be exposed. Similar environmental conditions and socioeconomic factors also likely determine the long-term risk of hantavirus exposure. Here, we use ecological niche models and human cases of HPS in the U.S. from 1993 to 2022 to assess hantavirus risk using four socioeconomic variables, 17 land use variables, one variable of rodent richness, and seven climate variables to determine both the geographical locations of highest exposure risk and leading environmental predictors. We found that areas with higher relative risk tend to be where it is drier, higher social vulnerability, increased rodent richness, and more open to low levels of development—this largely mapped to the western U.S. We found evidence that fringe ecosystems may be important areas of hantavirus transmission, similar to other emerging diseases. Increased rodent richness was associated with increased hantavirus risk, warranting further investigation into how the abundance and community composition of rodents could impact long-term risk. These risk maps can help public health officials develop plans for mitigating hantavirus, especially for the most susceptible populations. They can also be used to further investigate regions estimated to be at high risk for hantavirus where disease cases have not been as common but may be underreported.

## 1. Introduction

Hantaviruses (genus *Orthohantavirus*) are RNA viruses of the Hantaviridae family and can cause severe disease in humans. They are zoonotic in origin and primarily carried by rodents. In the Americas, hantaviruses can cause hantavirus pulmonary syndrome (HPS), which is an acute respiratory infection that has a mortality rate around 35% [1]. In the United States, Sin Nombre virus is the main etiological agent of HPS, and the primary reservoir is the deer mouse (*Peromyscus maniculatus*). Other variant strains of Sin Nombre virus that can cause HPS include Monongahela virus and New York orthohantavirus, the latter of which is harbored by the white-footed mouse (*Permyscus leucopus*) [2]. Bayou orthohantavirus and Black Creek Canal orthohantavirus are considered separate species and reservoir hosts are the rice rat (*Oryzomys palustris*) and the cotton rat (*Sigmodon hispidus*), respectively [2–4].

Hantaviruses were thought to be restricted to Asia and Europe until 1993, when an outbreak of unknown origin occurred in the Four Corners region of the U.S. In 1995, HPS became a nationally notifiable disease, and the national case definition was expanded in 2014 to include nonpulmonary hantavirus infections. As of the end of 2022, there have been 864 human cases of hantavirus reported in the U.S. [1], most of which were cases of HPS. Symptoms of HPS include fatigue, fever, muscle aches, and in the late stage of the disease, respiratory symptoms as lungs fill with fluid. Humans become infected with hantavirus from inhaling rodent feces and urine, and the disease is not spread from human to human. People can also become infected by touching their mouth or nose after handling contaminated materials. Although rare, being bitten by a rodent can also result in infection. The majority of hantavirus disease cases are caused by human exposure in their environment, whether that be their home, occupation, or through recreational activities [5].

The dynamics of hantavirus transmission are tied to both environmental and human-related factors. Interannual variations in climate, rodent populations, and seasonality are crucial for understanding annual disease risk and the potential for case outbreaks. However, estimating relative long-term risk across large geographic areas can help pinpoint regions where people are at high or low risk for infection regardless of environmental variability. To our knowledge, no study has examined the combined effects of climate, land use, socioeconomic factors, and rodent richness to determine hantavirus risk in the U.S. and identify variables most predictive of risk.

As main reservoir hosts, rodents are central to hantavirus risk. Modeling efforts to understand hantavirus epidemiology are usually done with rodent reservoir hosts and community dynamics as key parameters [6–8]. The majority of hantavirus cases in the U.S. are in the western half of the country and can be attributed to the Sin Nombre virus; the deer mouse (*P. maniculatus*) is the main reservoir host. This species is a generalist and can be found in many, if not all, habitat types that make up the western U.S. They are often the most abundant rodent species in many of these habitat types [9]. Different habitats and land use types support different densities and assemblages of hosts required for infection in humans [6, 10, 11]. The diversity of rodents may result in a dilution effect, where increased rodent diversity decreases the prevalence of hantavirus [12–16]. However, this may not always be the case—more research is needed to test this hypothesis and to understand the circumstances surrounding the opposite pattern, called the amplification effect [13, 17].

Land use can help describe where humans and rodents are interacting in suitable environments for hantavirus transmission. Studies on land use predictors of hantavirus infections have been done in the U.S., Latin America, and South America [18–20]. Most studies describe land use characteristics in relation to rodent population dynamics, such as the habitats that are suitable for rodent reservoirs [21]. Shrub and scrublands, including piñon-juniper woodlands, often have rodents with high seroprevalence of hantavirus antibodies [22] compared to woodland-riparian habitats [23]. These land use types are also where a greater proportion of humans live and recreate [24]. Confined spaces such as cabins and houses in poor condition are also a major source of infection, which are more common in rural areas [25]. However, deer mice also have high seroprevalence of hantavirus in disturbed habitats, including suburban and urban areas, increasing the risk where there are more people [23, 26, 27]. Conversely, deer mice are less likely to be found in city centers and in buildings, unless those buildings border woodlands or parks [28].

Climate conditions may also impact hantavirus transmission dynamics and likelihood of human infections. At interannual timescales, the density and abundance of rodent reservoir populations are tied to climate patterns, such as El Niño events and the resultant availability of food resources [29]. Climate on multidecadal timescales may also shape the relative risk of hantavirus. For example, temperatures and levels of environmental moisture may affect survival of the virus itself and impact the geographic distribution, population dynamics, and behavior of important rodent hosts [19, 30, 31]. Dry, dusty conditions, as are common in the western U.S., may cause increased exposure to infected rodent matter [5].

Human vulnerability may be driven by socioeconomic status and housing conditions [32, 33]. Studies find that poor sanitation leads to increased hantavirus risk, but these studies were done in Europe on a similar hantavirus-causing disease hemorrhagic fever with renal syndrome (HFRS) [34, 35]. In Brazil, an index of socioeconomic status, the Human Development Index (HDI), was positively correlated with HPS cases [33]. HDI measures human development and poverty and considers life expectancy, income, and education, with higher values indicating higher socioeconomic status. Unexpectedly, higher HDI was correlated with HPS risk. Higher risk was found in sugarcane plantations, which tend to have a higher abundance of rodents. Socioeconomic status is significantly higher in these plantations than surrounding areas [33], highlighting the complexity of environmental, socioeconomic, and rodent risk factors.

Previous spatial analyses of hantaviruses have established a strong foundation for ecological modeling using environmental and landscape predictors. In northeastern Arizona and northwestern New Mexico, spatial analyses were used to determine landscape and climate variables important for hantavirus infection risk, which included precipitation and piñon–juniper ecotones [36, 37]. Several studies demonstrate that hantavirus transmission is structured by predictable environmental and ecological patterns, and that long-term surveillance and predictive spatial modeling are essential tools for anticipating and mitigating human disease risk [29, 38]. Studies from Latin America and Eurasia have applied ecological niche modeling to map hantavirus risk zones, showing strong concordance between human cases and climate, vegetation, and host species distribution patterns [39, 40]. The emphasis on stable high-risk landscapes, consistent ecological correlates, and presence-only data on rodent hosts and human cases supports the rationale for predictive spatial models using land cover, climate, and topographic variables. We build on these spatial modeling analyses by applying Maxent modeling over a broad geographic scale to assess long-term hantavirus risk.

The overarching goal of our study was to assess hantavirus risk in the contiguous U.S. using many different predictor variables. Here, we define risk as the likelihood of an individual contracting hantavirus in a given location, agnostic of time. The risk of an individual contracting hantavirus can be thought of as the combined effects of the hazard (i.e., presence of hantavirus), human exposure, and vulnerability of that individual (e.g., sociodemographics) [41]. To combine the risk factors into a common assessment, we leveraged ecological niche modeling, which offers a statistical method to model geographic ranges for organisms using presence records. We adopt this tool to model the geographical range of hantavirus risk. Specifically, the ecological model we used is Maxent, which is well equipped to handle numerous environmental variables that may be spatially correlated [42, 43]. This approach also provides information on the relationships between each environmental variable and hantavirus risk, as well as how important each variable is for improving predictive accuracy. Together, this approach can provide information to public health officials on what populations are most at risk for contracting hantavirus and the potential drivers of disease risk so they can target appropriate disease mitigation strategies.

Specifically, our goals are to create maps to compare long-term risk between regions of the U.S. and to understand the spatial heterogeneity and primary variables associated with risk. Here, we use Maxent to assess hantavirus risk using four socioeconomic variables, 17 land use variables, one variable of rodent richness, and seven climate variables. We hypothesize that risk will be highest in areas with combinations of higher social vulnerability, shrub/scrub habitat, and human-disturbed land use (urban/suburban) because previous studies have identified high seroprevalence of hantavirus in rodents in these areas. Since most cases have occurred in the western U.S., we model risk in the entire U.S. and then run separate models on the western U.S. and eastern U.S. to avoid missing potentially important drivers in the eastern half of the country. We also sought to understand the contribution that different categories of variables would have in generating risk predictions. For this reason, we also ran models of each set of variables separately to help determine whether a certain category of variables can explain the majority of variation in hantavirus risk.

## 2. Methods

### 2.1. Hantavirus Case Data

We obtained human case data of hantavirus in the U.S. through a data use agreement with the U.S. Centers for Disease Control and Prevention (CDC). This dataset included domestic, annual case counts for the contiguous U.S. from 1993 to 2022. From the 864 total cases, five cases related to exposures outside of the U.S., seven associated with the Seoul virus outbreak, and 31 that occurred prior to 1993 were excluded. In total, this dataset included 821 cases. Apart from cases caused by the Sin Nombre virus, only 27 cases had information on the causative strain or orthohantavirus type. Of these, we removed 8 cases caused by Seoul virus from our dataset, since they were most likely tied to exposure from pet rats and not environmental exposure (*n* = 8, [44]). The year of illness onset was available for 811 of the cases. Where not available, we used the year of the patient case report (*n* = 2). Each case was coded as either HPS (*n* = 798), or hantavirus infection, nonpulmonary syndrome (*n* = 15). We only used cases of HPS, given that HPS cases are more severe disease and more likely to require healthcare and be captured by hantavirus surveillance networks. Our goal was to develop models based on the geographic location where infection occurred; therefore, we used cases that had exposure town (*n* = 584) information available. We converted the exposure town to latitude and longitude data using the Nominatim feature provided by Open Street Maps via the OSMPythonTools package (https://pypi.org/project/OSMPythonTools/) in Python (version 3.12). Some exposure information was incomplete, and we were not able to attribute it to a location. We had 575 cases with latitude and longitude identified at the town level (Figure S1).

To account for the clustering of cases, potential related outbreaks, and other spatial biases that may cause two or more cases to fall within the same environmental gridded pixel or overrepresent a particular spatial region, we spatially filtered the cases. We adjusted all latitude and longitude coordinates to 0.01° resolution, which is approximately equivalent to 1 km resolution. Then, we used the package spThin in R with an 8 km radial buffer so that only one case was used from random assignment within that 8 km radial buffer area [45, 46]. This reduces bias in the case dataset so that an outbreak of cases at a single location and an individual hantavirus case at a single location are weighted the same, since they both indicate suitability for hantavirus risk [47]. After filtering, we had 431 cases.

### 2.2. Environmental Data

We included data on climate, land use, socioeconomic, and rodent data as explanatory variables in our ecological niche models. We aggregated each gridded raster dataset from its native resolution to 4 km resolution using bilinear interpolation and matched the projections and spatial extents of the raster data. Our environmental data was at a finer resolution (4 km) than the hantavirus data (8 km), ensuring that only one hantavirus case fell within a single environmental grid cell since Maxent analyzes the occurrence record and the environmental grid cell as a coordinate pair.

To capture socioeconomic information, we used the U.S. Social Vulnerability Index (SVI) Grids (v1.01)[48]. This used four measures of vulnerability: socioeconomic, household composition, minority status, and housing type and transportation (Figure S2). Index values range from 0 to 1 based on their percentile position among all census tracts in the U.S., where 1 indicates the highest vulnerability. Though provided as a gridded raster, this data is intrinsically measured at the U.S. Census tract level to match its parent data from the U.S. Census Bureau. For this reason, there may be stark contrasts in vulnerability indices at U.S. Census tract borders. Despite this, we wanted to include information on socioeconomic status, since hantavirus risk may correlate with socioeconomic status [49]. The SVI was published in 2000, 2010, 2014, and 2020—we used the 2010 data to represent the median time period of our case data. Data for the SVI grids are unavailable in remote counties with no resident populations. As such, where SVI variables are used as predictors, the resultant hantavirus risk maps have areas where no risk is predicted and in the maps, grayed out.

We incorporated 16 different land cover types from the National Land Cover Database (NLCD) from the U.S. Geological Survey and Multi-Resolution Land Characteristics Consortium ([50, 51]; Figures S3,S4). We selected to use the 2011 data to represent the median time period of our case data. This categorical data has a native resolution of 30 m. We extracted each of the 16 binary (presence/absence) land use variables separately to calculate a fractional coverage estimate from 0 to 1 when coarsening the spatial resolution to match the other datasets. Though we acknowledge that land cover has changed over the course of our hantavirus case dataset, analyzing the fractional coverage (as opposed to a binary land cover classification) at an 8 km resolution should help to reduce any biases arising from land cover change by avoiding assigning only one land cover type to a hantavirus case.

We downloaded rodent richness data from BiodiversityMapping.org ([52]; Figure S5). Rodent richness is defined as the number of unique rodent species present in any given grid cell. Data were available on a 10 km grid. We converted this data to 4 km resolution using bilinear interpolation to match the other environmental variables. A list of the species incorporated in the rodent richness measure is included in the data download. The four main rodent hosts for hantavirus are included among the species in the map: *Peromyscus maniculatus*, *Peromyscus leucopus*, *Sigmodon hispidus*, and *Oryzomys palustris*. The original species range maps used to create the rodent richness maps were sourced from the International Union for Conservation of Nature (IUCN) in December 2017 [53].

We used seven climate variables from the TerraClimate dataset, which is available at a resolution of approximately 4 km ([54]; Figure S6). We included precipitation accumulation, minimum temperature, maximum temperature, snow water equivalent, and soil moisture. We calculated mean temperature by averaging minimum and maximum temperature values and temperature range by calculating the difference between the maximum and minimum temperatures. We averaged data from 1993 to 2022 to match the hantavirus case data.

We calculated the spatial correlations between each of the explanatory variables to assess collinearity. Pearson correlation values (*r*) between environmental variables ranged from −0.73 to 0.98. Particular classes of values exhibited multicollinearity; this included the social vulnerability variables (socioeconomic, household composition, minority status, and housing type and transportation; *r* = 0.30−0.74), temperature variables (minimum temperature, maximum temperature, mean temperature, temperature range; *r* = −0.05−0.98), and land use variables regarding level of development (developed, open space; developed, low intensity; developed, medium intensity; developed, high intensity; *r* = 0.36−0.82). Since there was no unexpected collinearity, we chose to not reduce our variables and instead consider these collinearities in the interpretation of our results (e.g., temperature may be an important predictor, though exactly which measure of temperature is less critical for interpreting results). Previous studies have shown that excluding highly correlated predictor variables does not significantly improve model performance since Maxent can regulate the contribution of redundant predictors [55].

### 2.3. Maxent Modeling

We used the machine learning, maximum entropy model Maxent to develop ecological niche models of hantavirus risk [42]. These methods have been previously described in part [56, 57, 58] and the methods description here partly reproduces the previously published wording. Here, since our hantavirus case data was present across the contiguous U.S., we did not subset an environmental training area but rather used the full extent of the contiguous U.S. We used the ENMevaluate package (version 2.0.0) in R [59, 60].

We used the 8 km filtered hantavirus case data and the 4 km gridded environmental data as input to the model. Using the entire contiguous U.S. as the environmental training area, we used Maxent to randomly generate 10,000 background points. Model training was conducted through cross-validation using five randomly assigned k-folds of the presence data. We evaluated three feature classes—linear (L), quadratic (Q), and hinge (H, also referred to as piecewise linear responses)—as well as their combinations. To assess model performance and complexity, we tested a range of regularization multipliers: 0.5, 1, 2, 5, 10, and 20 [61, 62]. In total, there were 42 competing models in the model selection process.

For our model selection procedure, we implemented a custom, previously published method using the ENMevaluate package with maxent.jar (v3.4.1) from the dismo package (v1.3.3; [56, 57, 63, 64, 58]. First, we filtered the models to retain only the half with the lowest absolute omission rate bias (avg.test.or10pct), following the approach of Pearson et al. [65]. Next, we applied an additional filter to select models with an average difference between training and testing AUC (avg.diff.AUC) below a defined threshold—the median AUC difference across all fitted models—calculated over the 5 k-fold cross-validation bins. Finally, from the remaining models, we chose the one with the lowest AICc value. For each environmental variable set (Table S1), we then extracted the variable-specific response curves from the top-performing Maxent model to interpret how each predictor was associated with hantavirus risk. For the top-performing model, we also obtained 10 model output replicates to assess the variability in our predictions [46, 66]. For the 10 replicates, we bootstrapped our data to use 80% of the hantavirus case data.

The maps produced were created using the mean habitat suitability among the 10 bootstrapped replicates. We normalized our models to the [0,1] range using the log–log transformation output, which can be generally interpreted as the suitability of hantavirus risk (i.e., contracting hantavirus; [43]). We label the model output as “low risk” at 0, “moderate risk” at 0.5, and “high risk” at 1. We subtracted the maximum and minimum habitat suitability among the 10 bootstrapped replicates as a measure of uncertainty (i.e., the range [0,1]) [66, 67].

We assessed the influence of each environmental variable in the Maxent models by examining the mean permutation importance averaged over the 10 bootstrapped models. A higher percentage indicates greater reliance of the model on that particular variable [42]. Although Maxent also provides percent contributions for each variable, these are determined heuristically based on the algorithm's specific path to a local optimum [68], and thus should be interpreted with caution.

In total, we created five hantavirus risk models: an all-variable risk model for the contiguous U.S., an all-variable model for the western U.S., an all-variable model for the eastern U.S., a social risk model (SVI variables only), a land feature risk model (land cover types and rodent richness variables only), and a climate risk model (climate variables only; Table 1).

## 3. Results

### 3.1. Hantavirus Cases

**Figure 1:**
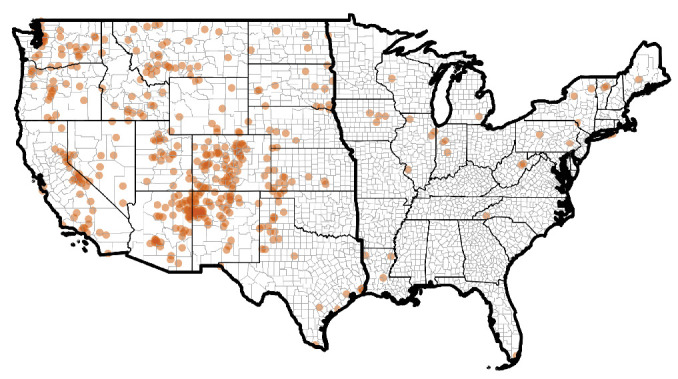
Hantavirus case reports used in our analysis after filtering and thinning the data (*n* = 431). Most of the cases occur in the western half of the U.S. (*n* = 400) compared to the eastern half (*n* = 31). As such, we ran two additional maxent models, separately analyzing the western and eastern halves of the country (thick black outlines).

After filtering and thinning the hantavirus case data, we used 431 case reports from 1993 to 2022 in our analysis (Figure 1). Most of these cases occurred in the western half of the U.S. (93%, *n* = 400) compared to the eastern half (7%, *n* = 31). Information on the particular species of hantavirus was only available for 14 cases: five were Bayou virus, five were Monongahela virus, three were New York virus, and one was Black Creek Canal virus. Due to this spatial bias in the distribution of cases, we created two additional maxent models using all covariates, separately analyzing the western and eastern halves of the country (thick black outlines in Figure 1). This additional analysis was to ensure that signals of disease risk in the eastern U.S. were not masked by important drivers of disease risk in the western U.S.

### 3.2. Hantavirus Risk Models

**Figure 2:**
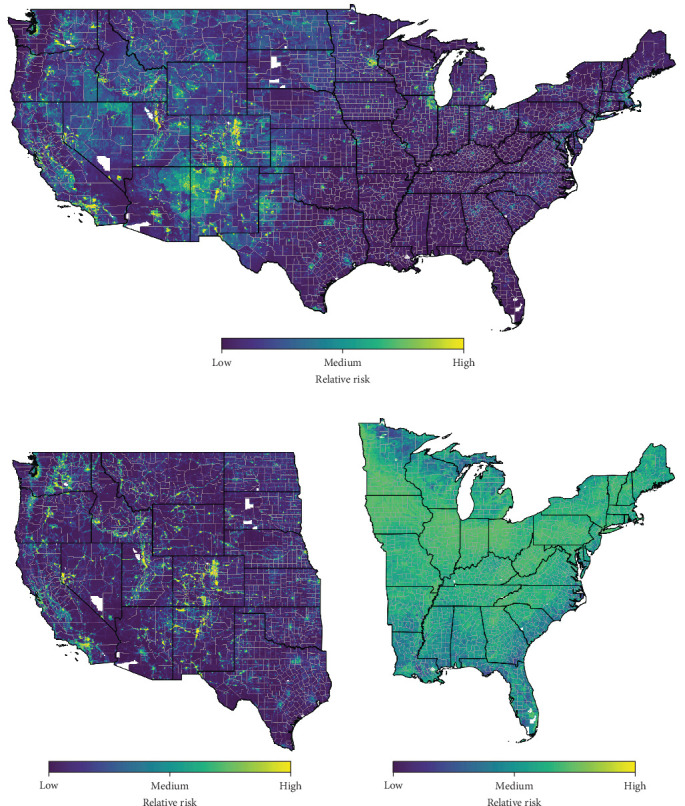
All-cause hantavirus risk maps for (a) the contiguous U.S., (b) the western U.S., and (c) the eastern U.S. Risk is output on a scale from 0 to 1, where 0 is labeled “low risk”, 0.5 is “moderate risk”, and 1.0 is “high risk”.

**Figure 3:**
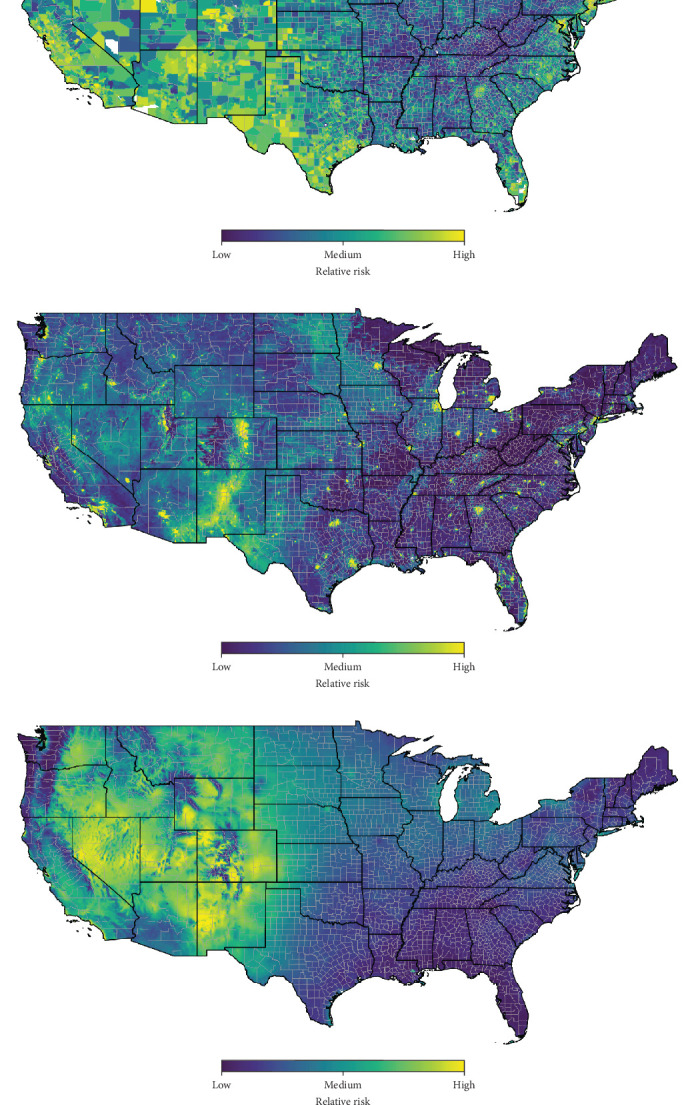
Themed hantavirus risk maps for the contiguous U.S. based on (a) social hantavirus risk; (b) land feature hantavirus risk; and (c) climate hantavirus risk. Risk is output on a scale from 0 to 1, where 0 is labeled “low risk,” 0.5 is “moderate risk,” and 1.0 is “high risk”.

Most models indicated the dry, western U.S. is at higher relative risk for hantavirus (Figures 2a,3). Areas with higher relative risk of hantavirus tend to be where it is drier, there is higher social vulnerability, increased rodent richness, and open to low levels of development. Though evergreen forests and shrubland covers were not very important variables for any of the hantavirus risk models, human cases of hantavirus seemed to be on fringe habitats (i.e., a transitional area between two different types of habitats) near these land types (Figure S4a,c). Each model slightly differed in model configuration (Table S1) and important variables structuring the spatial distribution of relative risk (Table 1 and Table S2).

For the contiguous U.S. all-variable model, the top three most important variables across all model iterations that account for almost half of the variable importance (48.2%) were developed, open space; developed, low intensity; and mean temperature (Table 1). Areas of highest risk are in central Colorado, northern New Mexico, Utah, southern California, eastern Washington, the Snake River Valley in Idaho, and throughout highway and interstate travel corridors and suburban areas in the western U.S. Interestingly, suburban areas around Chicago, Illinois; Detroit, Michigan; and Minneapolis, Minnesota have medium to high relative risk, though very few cases have been reported in these areas (Figures 1,S1). The eastern U.S. has relatively low risk for the all-variable contiguous U.S. model. The highest uncertainty in the model output for the all-variable hantavirus risk for the contiguous U.S. was in the Great Basin of Utah, the Rocky Mountains in Colorado, patchy regions across western Nevada and along the western coast of California, and across suburban areas in the central and midwestern U.S. (Figure S13a).

When separating the all-variable model into regional halves, the results and uncertainty for relative hantavirus risk for the western U.S. model were very similar to that of the all-variable model for the contiguous U.S. (Figure 2a,b; Figure S13b). The top three variables that accounted for 63.1% of variable importance were developed, open space; developed, low intensity; and mean temperature—the same as the contiguous U.S. model (Table 1; Figure S8). For the top model performance for the all-variable contiguous and western U.S. models, lower mean temperature was associated with higher risk (Figures S7,S8). For the contiguous U.S. model, the relationship between open and low-intensity developed land was parabolic, while the relationships for the western U.S. were hinged, so they were harder to directly compare.

The all-variable risk model for the eastern U.S. indicated there are different drivers of disease risk for the eastern U.S. than the western U.S. When analyzing the eastern U.S. alone, the levels of relative risk were rather homogeneously in the medium range (Figure 2c). Regions of relatively higher risk were in the northern half of the eastern U.S. The top important variables differed considerably from the western U.S. and contiguous U.S. all-variable models: woody wetlands, pasture/hay, and shrub/scrub and accounted for 88.1% of variable importance (Table 1). The top model performance suggested a negative relationship between hantavirus risk and pasture/hay, woody wetlands, and scrub/shrub (Figure S9). The relative risks in the suburban areas around Chicago, Detroit, and Minneapolis are no longer distinguishable from other surrounding locations like they were in the contiguous U.S. model. Likely driven by the relatively sparse case counts that this model was developed on (Figure 1), the uncertainty in the eastern U.S. model was much higher than the all-variable contiguous and western U.S. models (Figure S13c). Areas in the south and far northeastern U.S. were the most uncertain.

The social hantavirus risk model highlighted similar regions in the western U.S. as high relative risk, with some areas on the eastern seaboard with higher relative risk, too (Figure 3a). Out of the four SVI variables, household composition accounted for 46.1% of variable importance, followed by minority status at 25.8% and housing type and transportation at 19.8% (Table 1). Even when accounting for all other variables in the all-variable risk models for the western U.S., household composition accounted for 4.9% variable importance. No social variables were important for the eastern U.S. model. For the best-performing social risk model, there was a nonlinear positive relationship between household composition, minority status, socioeconomic status, and hantavirus risk (Figure S10). Interestingly, there was a nonlinear negative relationship between housing type and transportation. The relative uncertainty for the social hantavirus risk model was very low, especially considering that it only included four variables (Figure S14a).

The land feature risk model was similar to the contiguous U.S. all-variable risk model, highlighting how the land use variables were collectively the most important for structuring the spatial risk in the all-variable risk model. Together, rodent richness and deciduous forest were the two most important variables, accounting for 66.6% of variable importance (Table 1). Rodent richness was far less important in the other models, accounting for <1.0% of variable importance. The best-performing model suggests a nonlinear positive relationship between rodent richness and relative hantavirus risk and nonlinear negative relationship between deciduous forest and hantavirus risk (Figure S11). Many population centers are highlighted as relatively higher risk—each of the four land development variables had a positive relationship between development and predicted value of relative risk (Figure S11). The areas with the highest uncertainty are in Utah, southern Florida, and generally along the Rio Grande Rift Valley of New Mexico northward into Colorado (Figure S14b).

Lastly, the climate risk model again highlighted the dry, western U.S. as having the highest relative risk for hantavirus. The most important variable was precipitation (26.1%), while two measures of temperature were the next important—temperature range (23.5%) and maximum temperature (19.4%). The top-performing model suggests a negative relationship between precipitation and hantavirus risk (Figure S12). It also suggests the higher the maximum temperature, the lower the risk. Temperature range had a positive parabolic relationship with hantavirus risk—areas with high temperature ranges (likely, deserts; Figure S6) had the highest hantavirus risk. Many desert areas had higher levels of rodent richness, too (Figure S5). The areas with the highest uncertainty for the climate risk model were mostly in the extreme southwestern U.S., throughout California, and northern Idaho (Figure S14c).

## 4. Discussion

We used Maxent to assess long-term hantavirus risk using several socioeconomic, land use, rodent, and climate variables. Our question was: Can we understand the long-term risk of hantavirus using one category of variables or a set of key variables? We found that all four categories of variables were important for structuring the spatial risk of disease, though the land feature hantavirus risk model was most similar to the contiguous U.S. all-variable hantavirus risk model. Across the models, the most important variables were household composition; open and low development; deciduous forest; rodent richness; precipitation; and measures of temperature. Areas with the highest hantavirus risk are where it is drier, where there is higher social vulnerability, where there is increased rodent richness, and where there is open to low levels of development.

Our model results were more certain for the western U.S. than the eastern U.S., which is unsurprising given the majority of hantavirus cases are reported in the western U.S. Interestingly, the model for the eastern U.S. revealed important environmental drivers of disease that were different than the western U.S., including pasture/hay land cover, soil moisture, and woody wetlands. These results may largely be driven by the primary host species and species of hantavirus in this region. While cases of hantavirus in the western U.S. are predominantly attributed to Sin Nombre virus, numerous neglected orthohantaviruses are endemic to the eastern U.S., including Bayou virus, Black Creek Canal virus, Prospect Hill virus, Bloodland Lake virus, New York virus, Monongahela virus, and Blue River virus [69]. The Bayou orthohantavirus is carried by the marsh rice rat (*Oryzomys palustris*), which is a semiaquatic species in the southeastern U.S. that lives in wetland habitats such as swamps and salt marshes. This likely explains why woody wetlands land cover was an important variable for the eastern U.S. but not the western U.S. The Black Creek Canal orthohantavirus found in Florida is carried by the cotton rat (*Sigmodon hispidus*), which is less of a generalist compared to the common reservoirs for Sin Nombre virus (deer mice [*Peromyscus maniculatus*] and white-footed mice [*Permyscus leucopus*]). The cotton rat is found in pine, oak, grassland, and prairie environments throughout portions of the southern U.S. The cotton rat and rice rat are not found in outbuildings in rural areas where humans would also be dwelling [24], so it is likely that humans and rodents are not interacting in the same environments as frequently as humans and the reservoirs of Sin Nombre virus.

In general, the eastern U.S. is much wetter than the western U.S., which may play a key role in human exposure to infected rodent excrement. It is possible that the increased environmental moisture causes less rodent excrement to become aerosolized, reducing human exposure. More rainfall in the eastern U.S. may wash infected excrement away into storm drains, ditches, or other areas with limited risk of human exposure. Hantaviruses can survive in rodent excrement for up to 15 days [31], so the drier western U.S. may naturally accumulate more infected excrement in areas with human activity. Localized areas out of direct sunlight—areas likely preferred for sleeping by the nocturnal deer mice—may accumulate excrement that is viable for the longest period of time since hantaviruses are prone to desiccation [70].

Across several models, the response curves for multiple variables suggest that increased hantavirus risk may occur in fringe habitats. For the eastern U.S., this was evident with pasture/hay land cover and woody wetlands. Areas with proportions of pasture/hay below 0.4 were at much higher risk of hantavirus. Similarly, areas with less than 0.6 proportion of woody wetlands were at higher risk. In the western U.S., similar patterns were true for evergreen forests, shrubs, grasslands, and crops. This aligns with the understanding that areas of land-use change and human encroachment into wildlife habitat are areas of emerging pathogens [71, 72].

Across all models, household composition was the most important SVI variable. Household composition includes information on dependent children less than 18 years of age, persons aged 65 years and older, single-parent households, and people with disabilities. This was surprising given there is not much global evidence to suggest an age-stratified risk for contracting hantavirus and people less than 18 years of age in the U.S. only comprise about 10% of hantavirus cases [73–75]. This warrants future investigation to reassess the epidemiology of hantavirus in the U.S. and associated risk factors. There is, however, evidence to suggest homes with holes where rodents could enter and more rodents in the home contribute to higher hantavirus risk [34, 35, 76]. However, the housing type and transportation SVI index was not relatively important for determining the risk of hantavirus in our analysis. No measure of social vulnerability was important for the all-variable risk model in the eastern U.S., which may allude to the fact that rodents and humans are not interacting as much compared to the western U.S.

In the contiguous U.S. all-variable model, the western U.S. all-variable model, and the land use only model, rodent richness was positively associated with hantavirus risk. Though hantaviruses are typically thought to be associated with a primary host species, the positive association we found between rodent richness and hantavirus risk warrants further investigation to test whether hantaviruses can persist in multiple hosts with the same propensity [69]. Hantaviruses have also been recorded in moles and shrews in the U.S. [69, 77] and voles in Europe [34], demonstrating the ability of different species/strains to infect multiple hosts. Analyzing the same hantavirus species/strains among various potential hosts may provide insight into the interspecies spread of the virus. In the western U.S., where the Sin Nombre virus is dominant, increased rodent richness may be amplifying infection in deer mice and increasing human risk.

Recent evidence from rodent surveillance further highlights the complexity of hantavirus ecology. Sixteen rodent species in eastern New Mexico tested positive for Sin Nombre virus by qPCR [78]. Additionally, Astorga et al. [79] documented hantavirus seroprevalence across 15 rodent species—including six new hosts. Both studies reveal a broader range of potential reservoir species than traditionally considered. These findings support our results of rodent richness as a key variable in our risk models and emphasize the need to account for community-level host dynamics when modeling hantavirus risk.

Alternatively, the positive association with rodent richness is likely driven by the high number of cases in the Four Corners states, which have some of the highest rodent richness in the entire U.S. (Figure S5). Rodent richness was much more important in the land use only model than the all-variable models for the contiguous U.S. and western U.S.; despite Maxent itself robustly accounting for collinearity [55], this may indicate there are some underlying confounding effects between climate conditions and rodent richness. Rodent community-specific variables other than richness may provide a more appropriate measure of hantavirus prevalence independent of climate. Additional work should consider maps of density and abundance of rodent species that could impact transmission either by diluting the pathogen or amplifying infection risk. Thus, a community approach with rodent composition considered could uncover large-scale patterns across the western U.S. regarding which species, other than deer mice, are most influential.

Areas of low precipitation and higher temperatures were most important for structuring the spatial risk of hantavirus. This provides a baseline to speculate how hantavirus risk may shift in response to climate change. Though the magnitude of change is dependent on future greenhouse gas emissions, current projections of climate for the contiguous U.S. suggest the southwestern U.S. may be drier in the future, while the northwestern U.S. may become slightly wetter [80]. Coupled with projected increases in temperatures, this could exacerbate hantavirus risk in the southwestern U.S. The eastern U.S. is projected to become wetter, so hantavirus may remain primarily a disease endemic to the western U.S. Increased aridity in the western U.S. and dust storms [81] may increase human exposure to infectious excrement. Future land use and land cover change should also be considered in projections of hantavirus risk given the importance of the built environment and land use features in our models. Similarly, small projected increases in precipitation in Canada or slight decreases coupled with warming temperatures may increase hantavirus risk further north. Cases of HPS have been reported in Canada, and most cases are in the western part of the country in rural, agricultural areas [82]. As of January 2020, there have been 143 cases of HPS in Canada [82].

Current hantavirus risk is highest in underdeveloped areas (open to low levels of development) where human populations live in close contact with the natural habitats of rodents. We predict that habitat encroachment through low levels of development and transitional areas will be an important predictor of future risk. As humans encroach on wildlife habitats, there is a remarkable increase in the risk of zoonotic disease spillover to humans [83, 84]. Rodent populations are often displaced as land development increases, potentially reducing direct human–rodent contact in medium- and high-developed areas. In the short to medium term, the risk will likely be concentrated in underdeveloped or transitional areas rather than fully developed landscapes.

Despite open and low areas of development being associated with increased hantavirus risk, many population centers are depicted as relatively higher hantavirus risk in our maps. The hantavirus cases used to derive our models were geolocated to the likely case-exposure location, which is an advantage of this study over using cases geolocated to the location of disease diagnosis or case-patient residence. However, exposure location still introduces bias: hantavirus cases are more likely to occur where there is human activity. Our analysis mapped hantavirus risk as the likelihood of an individual contracting hantavirus in a given location, agnostic of time. Despite our efforts to reduce this bias by thinning the case data, we suspect we still see residual effects via our model output. How this bias may manifest is that areas of greater development (medium, high) may be positively related to hantavirus risk. Though the overall importances of the medium and high development land cover were low, the response curves still show a positive relationship for the all-variable, western U.S., and land feature models (Figures S7,S8,S11). To estimate the relative levels of disease cases or incidence, a different modeling approach including both spatial and temporal information of hantavirus cases and consideration of human population should be employed.

Based on our findings, future work assessing hantavirus risk should consider changes in land cover and fringe habitats. Our analysis was limited to the geographical precision of the town coordinates in which the person was likely exposed, which may introduce some bias into our analysis since exact exposure location is hard to determine. Further work could consider using genomic analyses to link environmental exposure to disease cases. We lumped all cases of HPS together regardless of the virus type since we did not have this information. Further disease surveillance efforts to define the causative orthohantavirus will help elucidate differences in disease risk across the viruses.

## 5. Conclusions

We use ecological niche models and human cases of HPS to assess hantavirus risk in the U.S. Areas where it is dry, there is increased higher social vulnerability, increased rodent richness, and more open to low levels of development had higher hantavirus risk. Risk was higher in the western U.S. than the eastern U.S. We found evidence that fringe ecosystems may impact hantavirus transmission, similar to other emerging diseases. Increased rodent richness was positively correlated with increased hantavirus risk, warranting further investigation into rodent community-specific measures to assess risk. Coupling long-term disease risk assessments like we have done here with mechanistic models of interannual disease risk, including variables like deer mice density, is an important next step for a holistic view of hantavirus risk. From a public health perspective, the all-variable risk map best represents the risk of hantavirus in the U.S. However, all risk maps that were created can help public health officials develop plans for mitigating disease for the most susceptible populations, especially in the western U.S. They can also be used to further investigate regions estimated to be at high risk for hantavirus where disease cases have not been reported. Projecting hantavirus risk according to different climate change scenarios will be critical to identify areas not historically impacted by infections and outbreaks.

## Figures and Tables

**Table 1:** Mean percent permutation importance of each environmental variable averaged across the 10 bootstraps of the top model of hantavirus risk.

Category	Variable	All-variable risk: contiguous U.S.	All-variable risk: western U.S.	All-variable risk: eastern U.S.	Social risk	Land feature risk	Climate risk
Social vulnerability index	Socioeconomic	1.3	0.3	0.0	8.3	—	—
	Household composition	2.3	4.9	0.0	46.1	—	—
	Minority status	0.2	0.1	0.0	25.8	—	—
	Housing type and transportation	0.9	1.8	0.0	19.8	—	—

NLCD land cover	Water	0.2	0.1	0.0	—	0.7	—
	Perennial ice/snow	0.0	0.0	0.0	—	0.0	—
	Developed, open space	19.2	28.3	0.0	—	10.9	—
	Developed, low intensity	15.7	24.6	0.0	—	3.4	—
	Developed, medium intensity	0.0	3.0	0.0	—	6.4	—
	Developed, high intensity	0.3	2.6	0.0	—	0.3	—
	Barren land	0.2	0.0	0.0	—	0.2	—
	Deciduous forest	11.1	0.5	0.0	—	29.8	—
	Evergreen forest	0.9	0.3	0.0	—	0.1	—
	Mixed forest	0.0	0.0	0.0	—	0.7	—
	Shrub/scrub	8.6	1.1	16.3	—	0.1	—
	Grassland/herbaceous	7.5	3.9	0.0	—	2.4	—
	Pasture/hay	0.0	0.0	29.0	—	1.4	—
	Cultivated crops	0.9	3.2	0.0	—	1.5	—
	Woody wetlands	1.0	0.0	42.8	—	4.2	—
	Emergent herbaceous wetlands	0.0	0.0	1.4	—	0.9	—

Rodents	Rodent richness	0.2	0.4	0.0	—	36.8	—

Climate	Precipitation	10.1	1.5	0.0	—	—	26.1
	Minimum temperature	0.0	8.2	0.0	—	—	13.4
	Maximum temperature	0.1	0.0	0.0	—	—	19.4
	Mean temperature	13.3	10.2	0.0	—	—	16.7
	Temperature range	5.4	3.5	0.0	—	—	23.5
	Snow water equivalent	0.1	0.3	0.0	—	—	0.1
	Soil moisture	0.3	1.0	10.5	—	—	0.8

*Note:* Cells without a value means that the variable was not included in the model selection process.

## Data Availability

The hantavirus data that support the findings of this study are available upon request through a data use agreement from the U.S. Centers for Disease Control and Prevention. The data are not publicly available due to patient privacy restrictions.

## Supporting Information

Supporting Information An additional Supporting Information file contains Tables S1–S2 and Figures S1–S14. Table S1. Final specifications and performance metrics for each risk model. Table S2. Mean percent variable contribution of each environmental variable averaged across the ten10 bootstraps of the top model of hantavirus risk. Figure S1. Hantavirus case reports before filtering. There were 575 cases from 1993 to 2022 that had latitude and longitude identified at the town-level. Figure S2. Four social risk variables included in the all-variable and social risk models from the U.S. Social Vulnerability Index Grids. Index values range from 0 to 1 based on their percentile position among all census tracts in the US, where 1 indicates the highest vulnerability. The four subsets of vulnerability are: (a) socioeconomic, (b) household composition, (c) minority status, and (d) housing type and transportation. Figure S3. First set of eight of the sixteen16 fractional land cover and land use variables (range 0–1) from the NLCD included in the all-variable and land feature risk models, including (a) water, (b) perennial ice/snow, (c) developed, open space, (d) developed, low intensity, (e) developed, medium intensity, (f) developed, high intensity, (g) barren land, and (h) deciduous forest. Figure S4. Second set of eight of the sixteen16 fractional land cover and land use variables (range 0–1) from the NLCD included in the all-variable and land feature risk models, including (a) evergreen forest, (b) mixed forest, (c) shrub/scrub, (d) grassland/herbaceous, (e) pasture/hay, (f) cultivated crops, (g) woody wetlands, and (h) emergent herbaceous wetlands. Figure S5. Rodent richness included in the all-variable and land feature risk models. Figure S6. Seven climate risk variables included in the all-variable and (a) precipitation, (b) minimum temperature, (c) maximum temperature, (d) mean temperature, (e) temperature range, (f) snow water equivalent, and (g) soil moisture. Figure S7. Response curves for the contiguous U.S. all-variable hantavirus risk model. Social Vulnerability Indices are 0–1, land use/cover variables are fractional values from 0 to 1, and the climate variables and rodent richness are on scales unique to that variable. Figures S8. Response curves for the all-variable: western U.S. hantavirus risk model. Social Vulnerability Indices are 0–1, land use/cover variables are fractional values from 0 to 1, and the climate variables and rodent richness are on scales unique to that variable. Figure S9. Response curves for the all-variable: eastern U.S. hantavirus risk model. Social Vulnerability Indices are 0–1, land use/cover variables are fractional values from 0 to 1, and the climate variables and rodent richness are on scales unique to that variable. Figure S10. Response curves for the social hantavirus risk model. Social Vulnerability Indices are 0–1. Figure S11. Response curves for the land feature hantavirus risk model. Land use/cover variables are fractional values from 0 to 1. Rodent richness is on a unique scale. Figure S12. Response curves for the climate hantavirus risk model. Each variable is on a scale unique to that variable. Figure S13. Maps of the range [0–1] between the maximum and minimum all-variable hantavirus risk amongst the ten10 bootstrapped replicates to show areas of high or low uncertainty for (a) the contiguous U.S., (b) the western U.S., and (c) the eastern U.S. We label the range as such: 0.00 is “low uncertainty,” 0.25 is “moderate uncertainty,” and 0.50 is “high uncertainty.” Figure S14. Maps of the range [0–1] between the maximum and minimum hantavirus risk models amongst the ten10 bootstrapped replicates to show areas of high or low uncertainty for the (a) social risk model, (b) land feature risk model, and (c) climate risk model. We label the range as such: 0.00 is “low uncertainty,” 0.25 is “moderate uncertainty,” and 0.50 is “high uncertainty.”
