# Supplementary material for: Hantavirus is Associated With Open Developed Areas and Arid Climates, Highlighting Increased Risk in the Western United States
Source: Transbound Emerg Dis. 2025 Oct 16;2025:7126411. doi: 10.1155/tbed/7126411 (PMC12549197; doi:10.1155/tbed/7126411)

***Supporting Information***

**Hantavirus is associated with open developed areas and arid climates, highlighting increased risk in the western United States**

Morgan E. Gorris^1^, Amy Whitesell^2^, Carson Telford^2^, Trevor Shoemaker^2^, and Andrew W. Bartlow^3^

^1^Information Systems and Modeling, Los Alamos National Laboratory, Los Alamos, NM, USA

^2^Viral Special Pathogens Branch, U.S. Centers for Disease Control and Prevention, Atlanta, GA, USA

^3^Genomics and Bioanalytics, Los Alamos National Laboratory, Los Alamos, NM, USA

*Corresponding author: Morgan Gorris, mgorris@lanl.gov

**Table S1.** Final specifications and performance metrics for each risk model.

|  | Feature classes | 𝛌 | Train AUC | AUC Val. | AUC Diff. | Mean OR 10% | SD OR 10% | AICc | ΔAICc | # of parameters |
| --- | --- | --- | --- | --- | --- | --- | --- | --- | --- | --- |
| *All-variable risk: contiguous U.S.* | LQ | 2 | 0.91 | 0.90 | 0.011 | 0.114 | 0.017 | 9847 | 185 | 25 |
| *All-variable risk: western U.S.* | LQH | 5 | 0.94 | 0.93 | 0.012 | 0.110 | 0.042 | 8589 | 0 | 64 |
| *All-variable risk: eastern U.S.* | L | 10 | 0.84 | 0.74 | 0.105 | 0.195 | 0.184 | 728 | 0 | 7 |
| *Social risk* | L | 1 | 0.71 | 0.71 | 0.020 | 0.102 | 0.039 | 11024 | 17 | 4 |
| *Land feature risk* | L | 0.5 | 0.85 | 0.84 | 0.010 | 0.112 | 0.018 | 10453 | 537 | 15 |
| *Climate risk* | LQ | 0.5 | 0.76 | 0.75 | 0.018 | 0.105 | 0.008 | 10897 | 0 | 10 |

*Notes:*

**<Table name** **(variable name in ENMeval):** definition>

**Feature classes (fc):** denoted as L for linear function, Q for quadratic function, and H for hinge function

𝛌 (rm): Regularization multiplier

**Train AUC (auc.train):** Area under the curve (AUC) calculated on the full dataset

**AUC Val. (auc.val.avg):** average AUC calculated on the validation datasets (the data withheld during cross-validation)

**AUC Diff. (auc.diff.avg):** average difference between auc.train and auc.val

**Mean Test OR 10% (or.10p.avg):** average omission rate (OR) with threshold as the minimum suitability value across occurrence records after removing the lowest 10%

**SD OR 10% (or.10p.sd):** standard deviation of the omission rate with threshold as the minimum suitability value across occurrence records after removing the lowest 10%

**AICc (AICc):** AIC corrected for small sample sizes

**ΔAICc (delta.AICc):** highest AICc value across all models minus this model's AICc value, where lower values mean higher performance and 0 is the highest performing model

**# of parameters:** number of non-zero beta values (model coefficients)

# **Table S2.** Mean percent variable contribution of each environmental variable averaged across the ten bootstraps of the top model of hantavirus risk.

| **Category** | **Variable** | **All-variable risk: Contiguous U.S.** | **All-variable risk: Western U.S.** | **All-variable risk: Eastern U.S.** | **Social risk** | **Land feature risk** | **Climate risk** |
| --- | --- | --- | --- | --- | --- | --- | --- |
| **Social vulnerability index** | **Socioeconomic** | 1.3 | 0.1 | 0.0 | 4.2 |  |  |
|  | **Household Composition** | 5.9 | 2.3 | 0.0 | 67.0 |  |  |
|  | **Minority Status** | 0.8 | 0.0 | 0.0 | 10.8 |  |  |
|  | **Housing Type and Transportation** | 0.1 | 2.1 | 0.0 | 18.1 |  |  |
| **NLCD  Land cover** | **Water** | 0.1 | 0.0 | 0.0 |  | 0.3 |  |
|  | **Perennial ice/snow** | 0.0 | 0.0 | 0.0 |  | 0.0 |  |
|  | **Developed, open space** | 8.0 | 4.8 | 0.0 |  | 4.0 |  |
|  | **Developed, low intensity** | 24.4 | 41.5 | 0.0 |  | 22.3 |  |
|  | **Developed, medium intensity** | 15.3 | 16.3 | 0.0 |  | 23.3 |  |
|  | **Developed, high intensity** | 0.1 | 11.5 | 0.0 |  | 0.2 |  |
|  | **Barren land** | 0.2 | 0.0 | 0.0 |  | 0.2 |  |
|  | **Deciduous forest** | 6.0 | 0.2 | 0.0 |  | 14.8 |  |
|  | **Evergreen forest** | 0.6 | 0.4 | 0.0 |  | 0.0 |  |
|  | **Mixed forest** | 0.2 | 0.0 | 0.0 |  | 0.5 |  |
|  | **Shrub/Scrub** | 2.5 | 2.0 | 12.1 |  | 0.1 |  |
|  | **Grassland/ Herbaceous** | 3.0 | 1.1 | 0.0 |  | 1.3 |  |
|  | **Pasture/hay** | 0.1 | 0.0 | 6.5 |  | 0.4 |  |
|  | **Cultivated crops** | 0.3 | 0.5 | 0.0 |  | 0.9 |  |
|  | **Woody wetlands** | 0.2 | 0.0 | 72.3 |  | 1.7 |  |
|  | **Emergent herbaceous wetlands** | 0.0 | 0.0 | 2.3 |  | 0.6 |  |
| **Rodents** | **Rodent richness** | 3.5 | 0.4 | 0.0 |  | 29.4 |  |
| **Climate** | **Precipitation** | 2.8 | 0.2 | 0.0 |  |  | 31.4 |
|  | **Minimum temperature** | 7.8 | 3.7 | 0.0 |  |  | 12.6 |
|  | **Maximum temperature** | 0.1 | 0.0 | 0.0 |  |  | 0.9 |
|  | **Mean temperature** | 1.6 | 0.7 | 0.0 |  |  | 2.7 |
|  | **Temperature range** | 12.8 | 1.9 | 0.0 |  |  | 28.0 |
|  | **Snow water equivalent** | 0.0 | 6.6 | 0.0 |  |  | 2.2 |
|  | **Soil moisture** | 2.4 | 3.6 | 6.8 |  |  | 22.2 |

**Figure S1.** Hantavirus case reports before filtering. There were 575 cases from 1993-2022 that had latitude and longitude identified at the town-level.


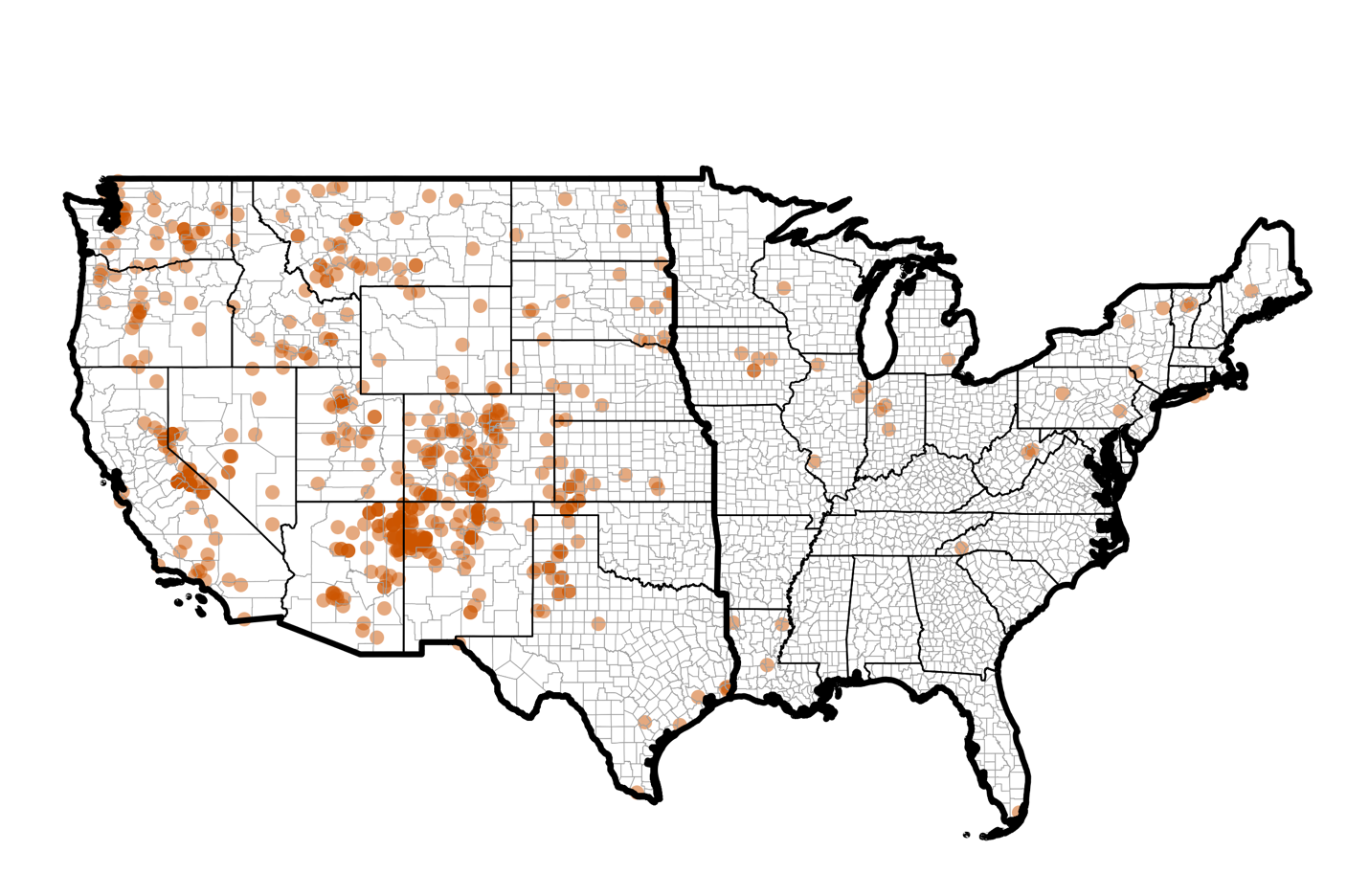


**Figure S2.** Four social risk variables included in the all-variable and social risk models from the US Social Vulnerability Index Grids (CDC, 2024b). Index values range from 0 to 1 based on their percentile position among all census tracts in the US, where 1 indicates the highest vulnerability. The four subsets of vulnerability are: a) socioeconomic, b) household composition, c) minority status, and d) housing type and transportation.

**
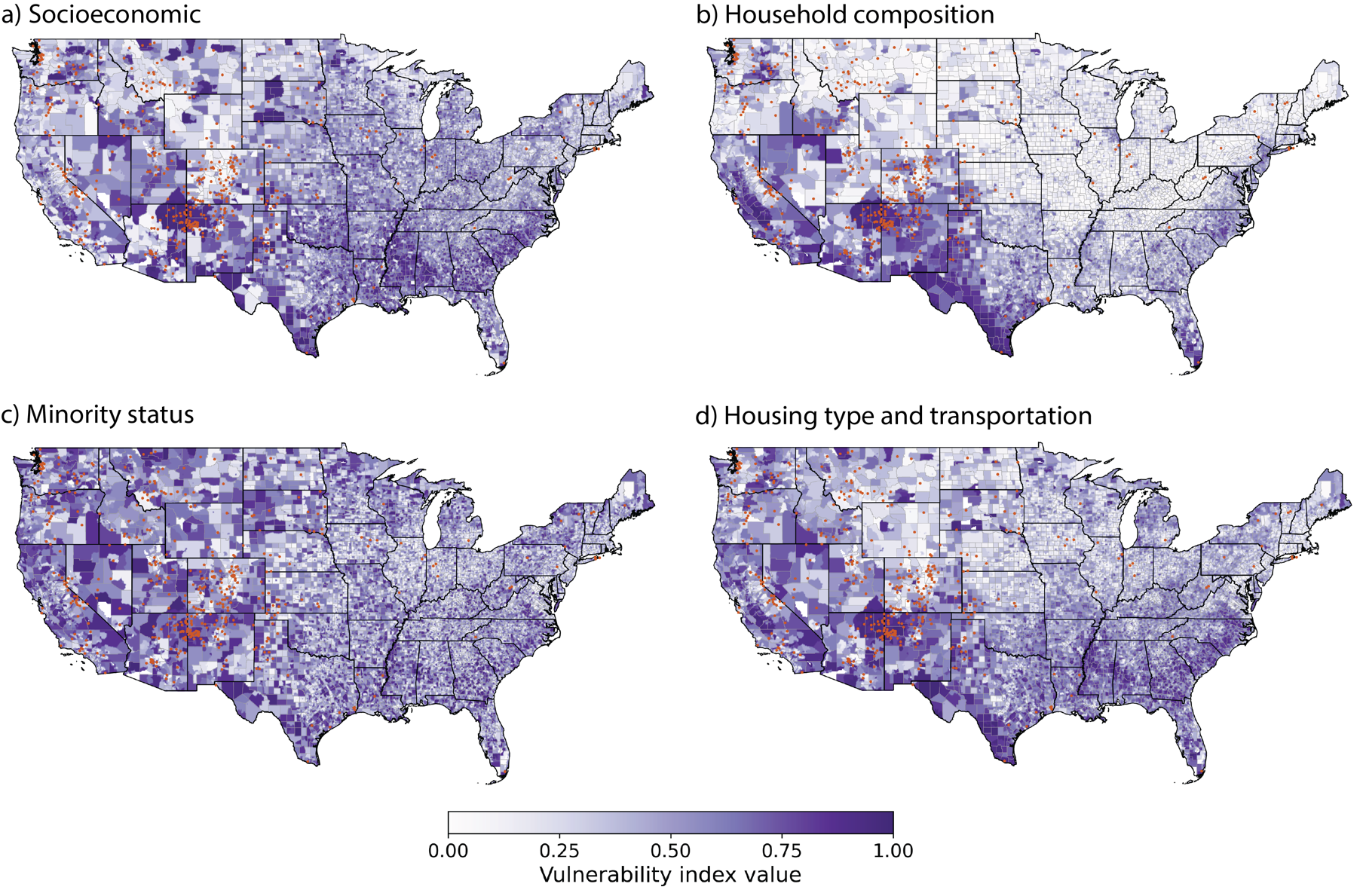
**

**Figure S3.** First set of eight of the sixteen fractional land cover and land use variables (range 0-1) from the NLCD (Homer et al., 2015; Multi-Resolution Land Characteristics Consortium, 2011) included in the all-variable and land feature risk models, including a) water, b) perennial ice/snow, c) developed, open space, d) developed, low intensity, e) developed, medium intensity, f) developed, high intensity, g) barren land, and h) deciduous forest.


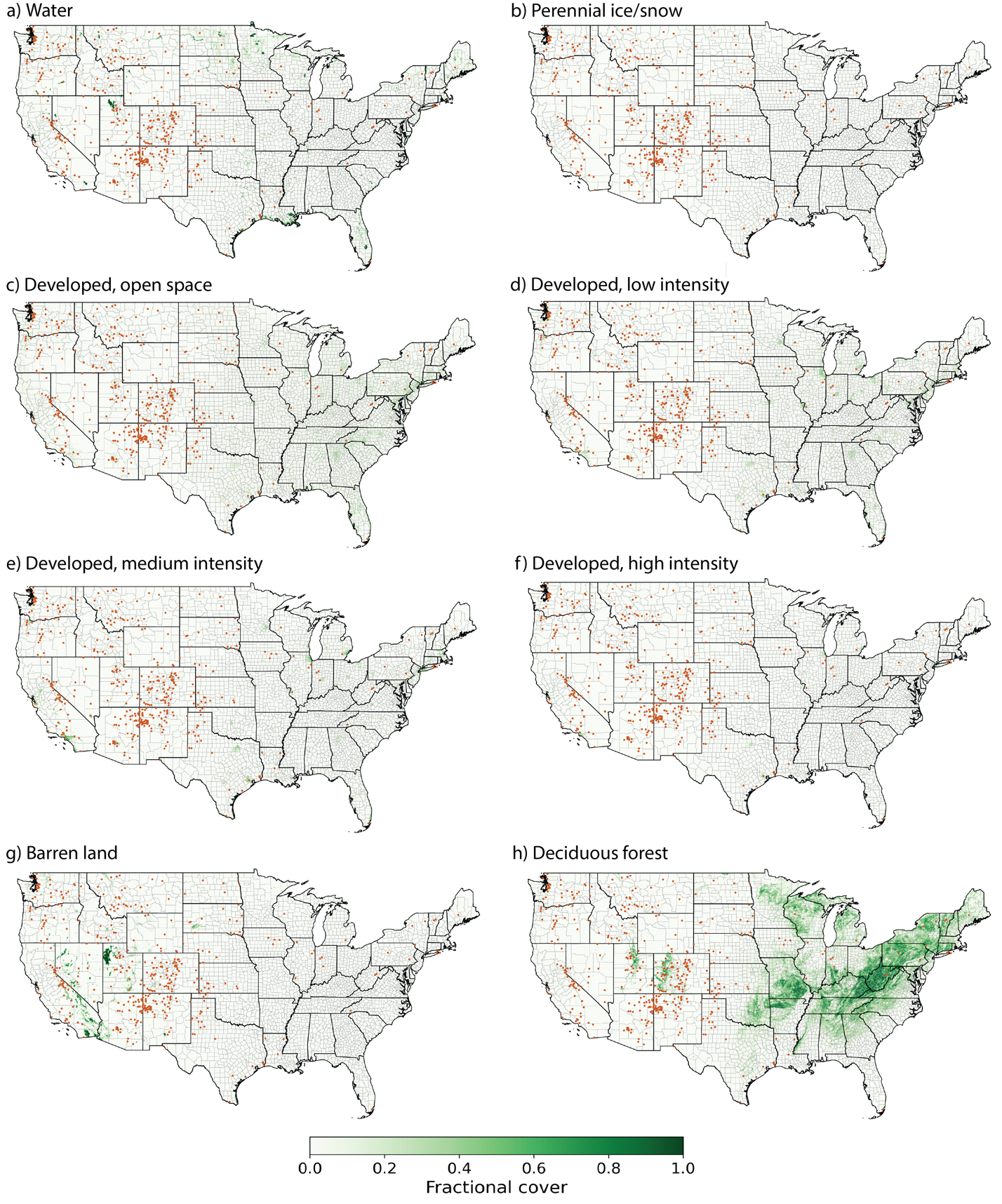


**Figure S4.** Second set of eight of the sixteen fractional land cover and land use variables (range 0-1) from the NLCD (Homer et al., 2015; Multi-Resolution Land Characteristics Consortium, 2011) included in the all-variable and land feature risk models, including a) evergreen forest, b) mixed forest, c) shrub/scrub, d) grassland/herbaceous, e) pasture/hay, f) cultivated crops, g) woody wetlands, and h) emergent herbaceous wetlands.


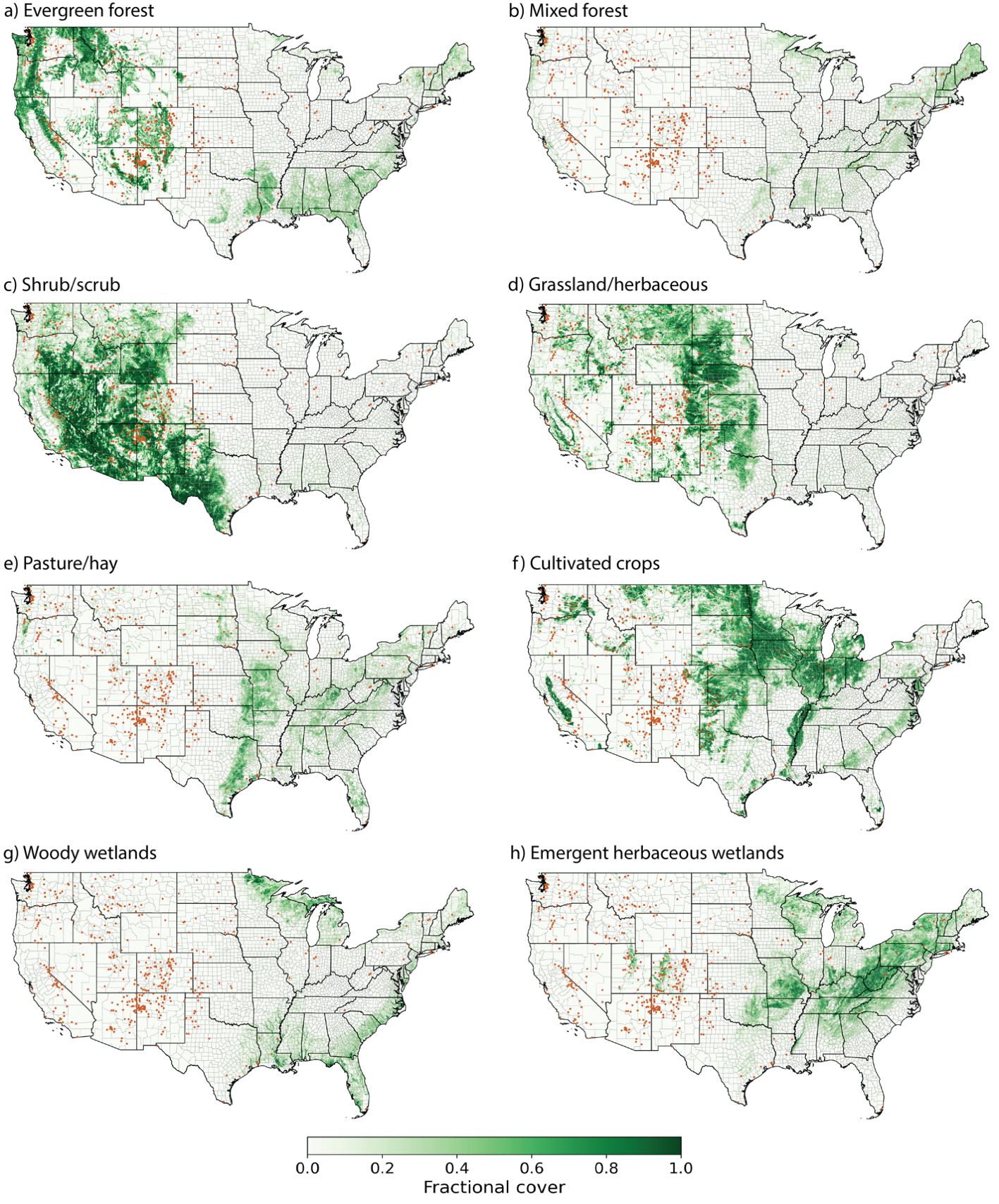


**Figure S5.** Rodent richness from BiodiversityMapping.org (Jenkins et al., 2015) included in the all-variable and land feature risk models.

**
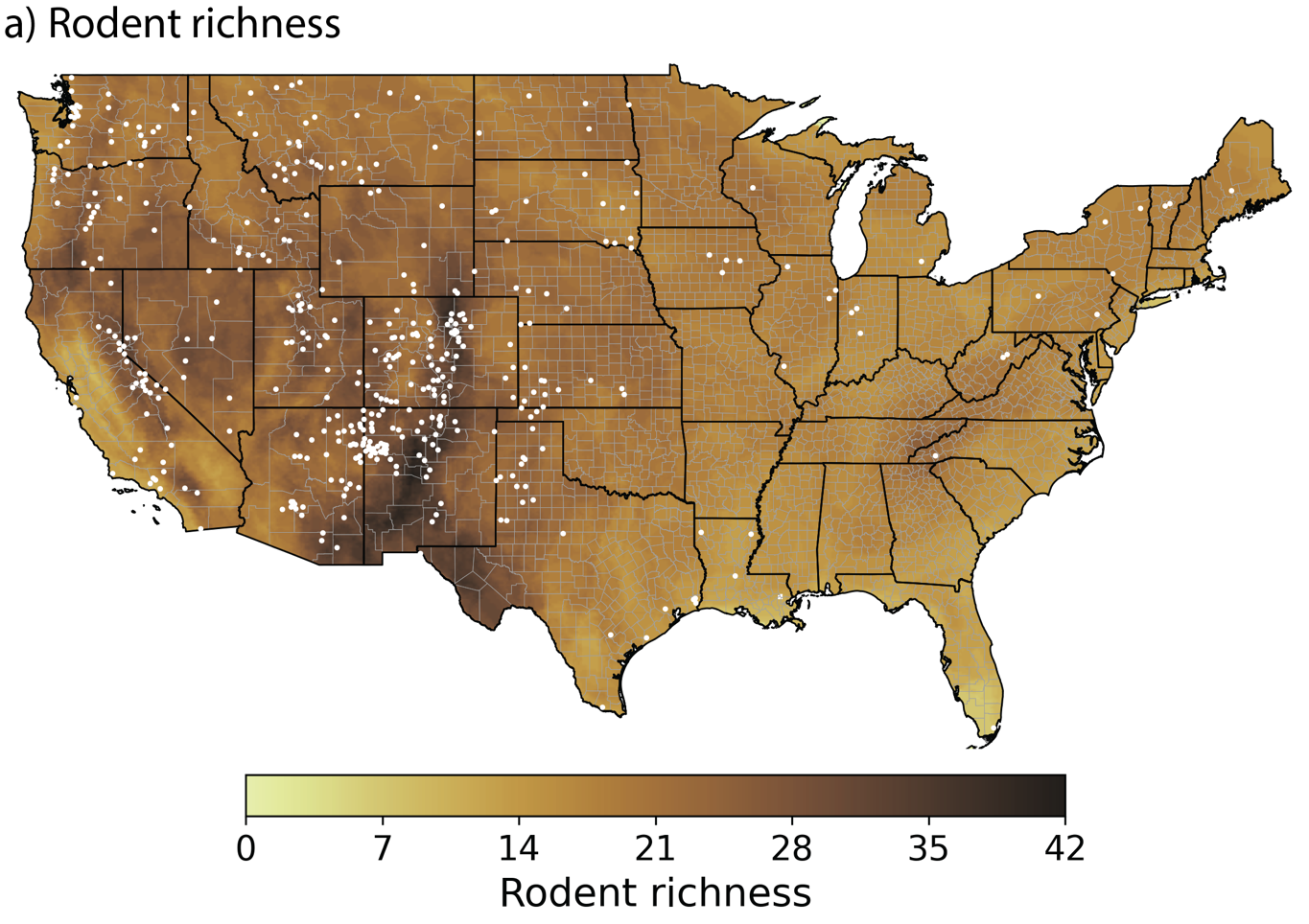
**

**Figure S6.** Seven climate risk variables from TerraClimate (Abatzoglou et al., 2018) included in the all-variable and a) precipitation, b) minimum temperature, c) maximum temperature, d) mean temperature, e) temperature range, f) snow water equivalent, and g) soil moisture.


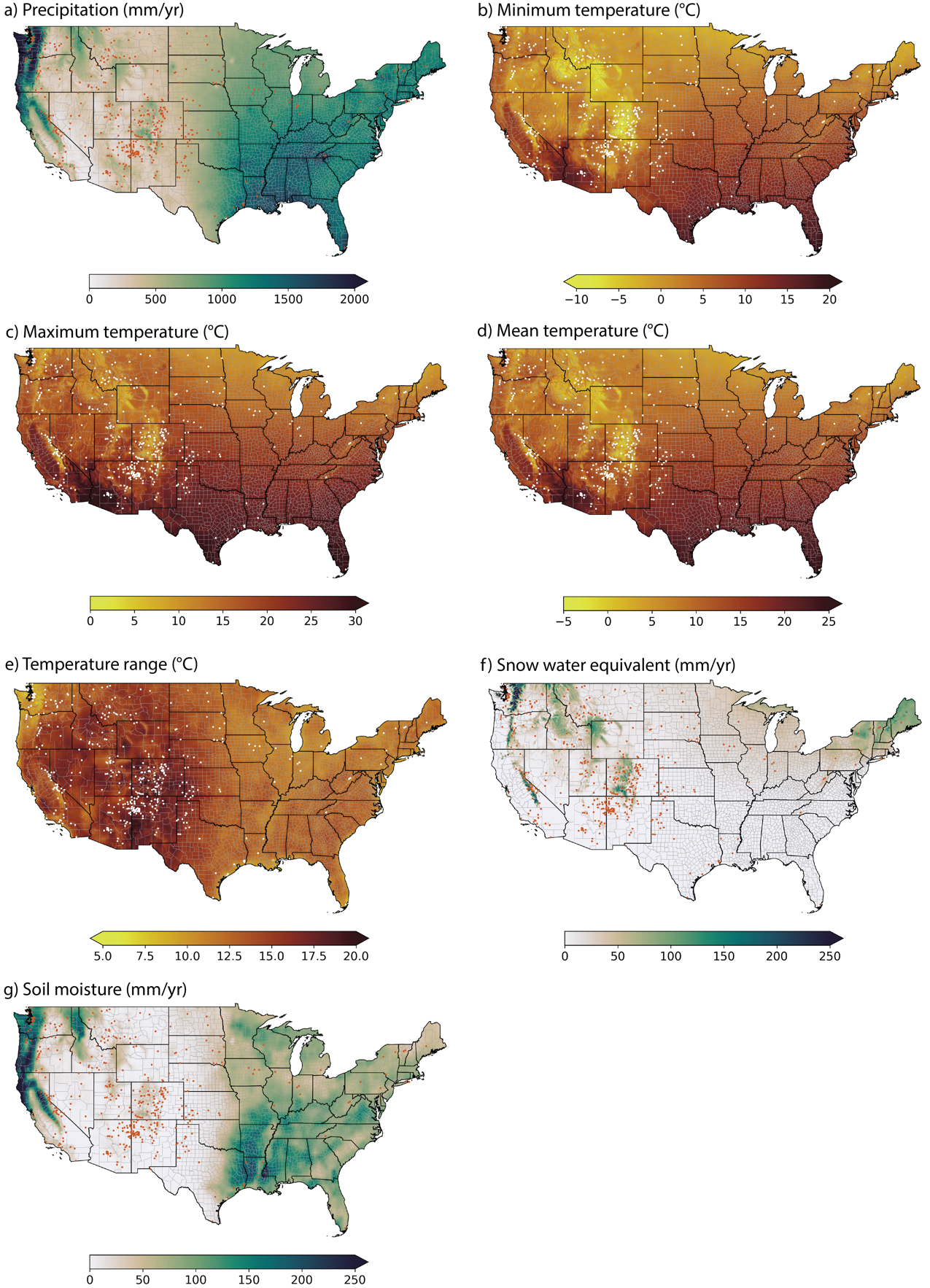


**Figure S7.** Response curves for the contiguous U.S. all-variable hantavirus risk model. Social Vulnerability Indices are 0-1, land use/cover variables are fractional values from 0-1, and the climate variables and rodent richness are on scales unique to that variable.

**
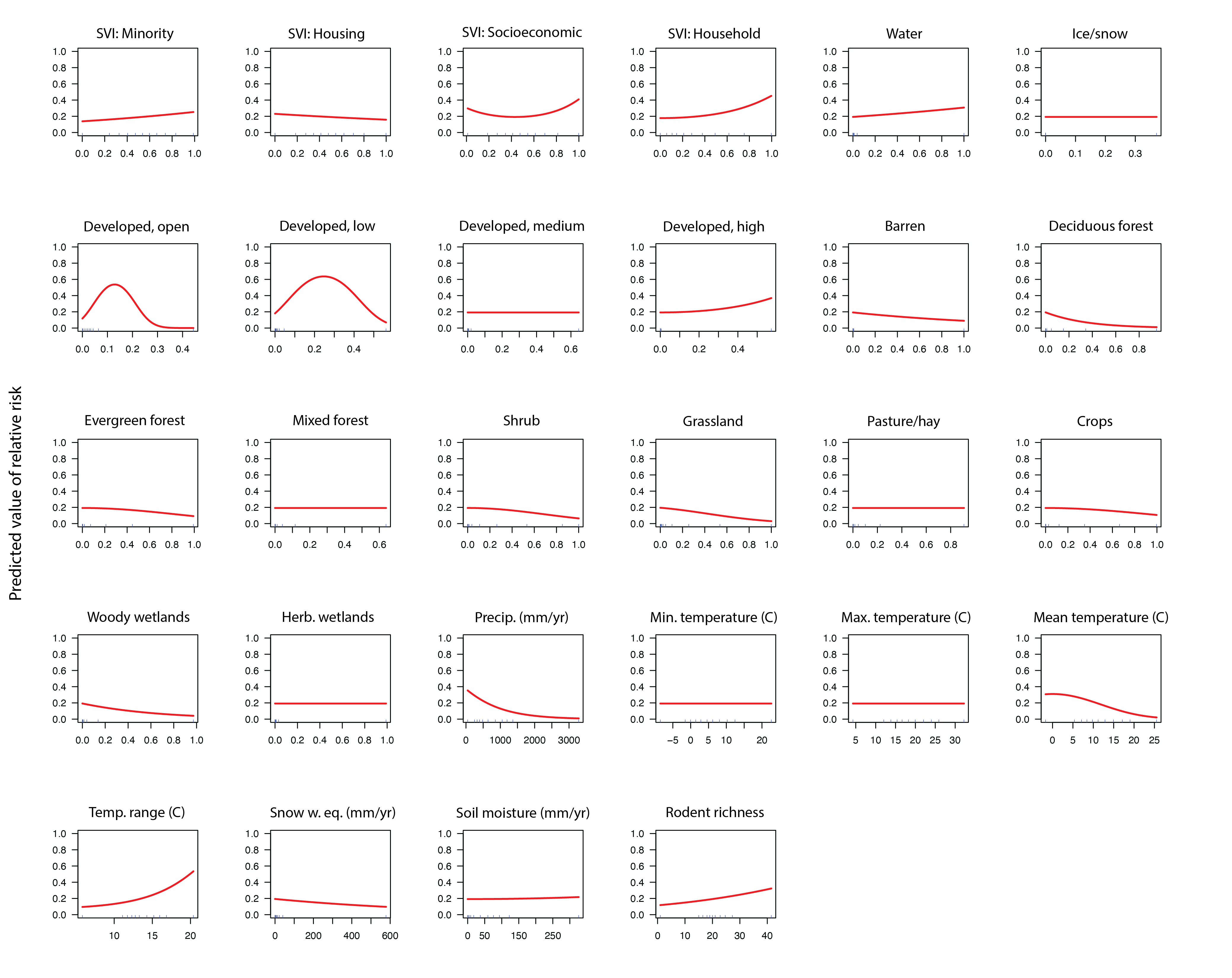
**

*Note:* SVI is Social Vulnerability Index; herb. is herbaceous; precip. is precipitation; min. is minimum; temp. is temperature; max. is maximum; w. is water; eq. is equivalent; m. is moisture.

**Figures S8.** Response curves for the all-variable: western U.S. hantavirus risk model. Social Vulnerability Indices are 0-1, land use/cover variables are fractional values from 0-1, and the climate variables and rodent richness are on scales unique to that variable.

**
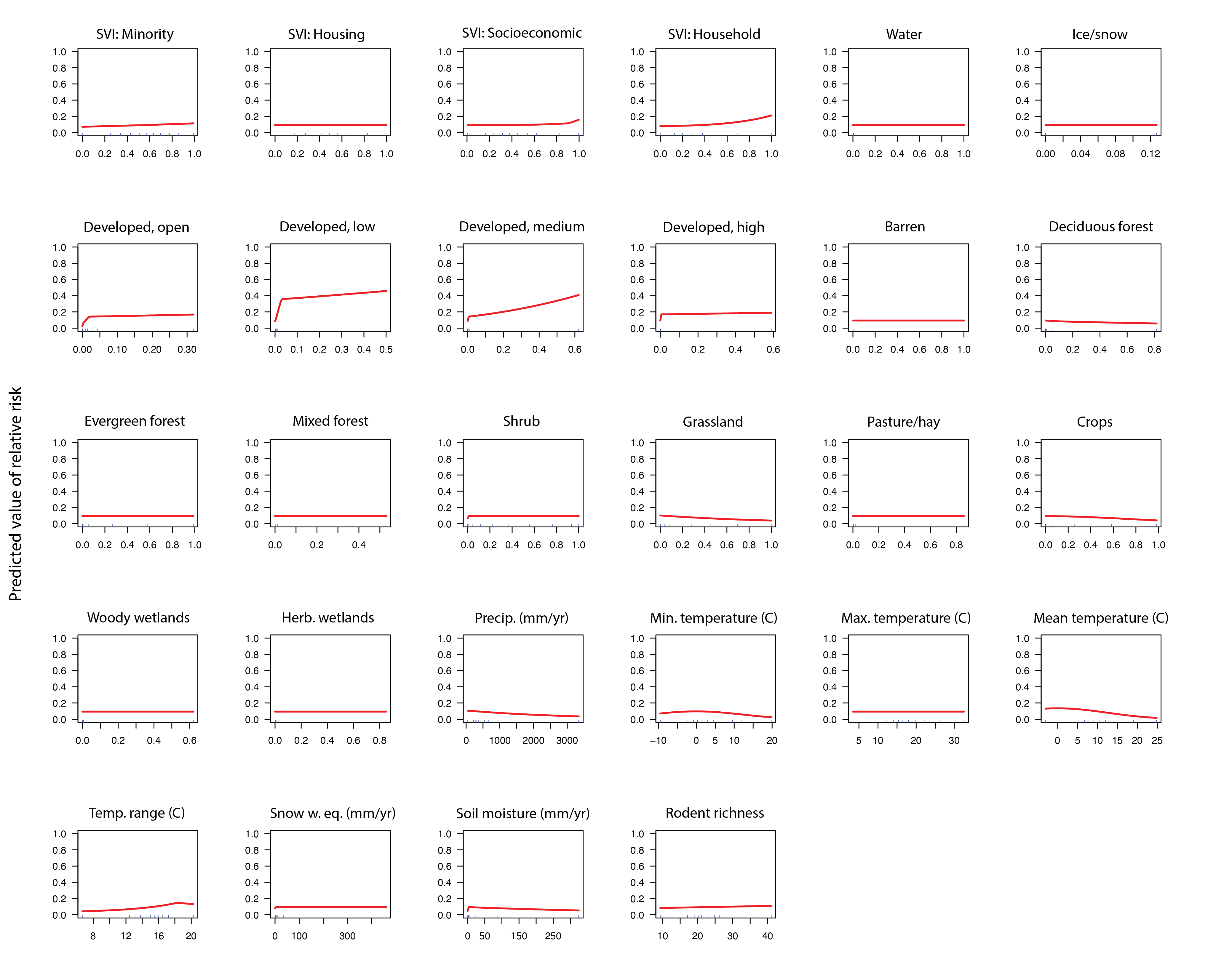
**

*Note:* SVI is Social Vulnerability Index; herb. is herbaceous; precip. is precipitation; min. is minimum; temp. is temperature; max. is maximum; w. is water; eq. is equivalent; m. is moisture.

**Figure S9.** Response curves for the all-variable: eastern U.S. hantavirus risk model. Social Vulnerability Indices are 0-1, land use/cover variables are fractional values from 0-1, and the climate variables and rodent richness are on scales unique to that variable.

**
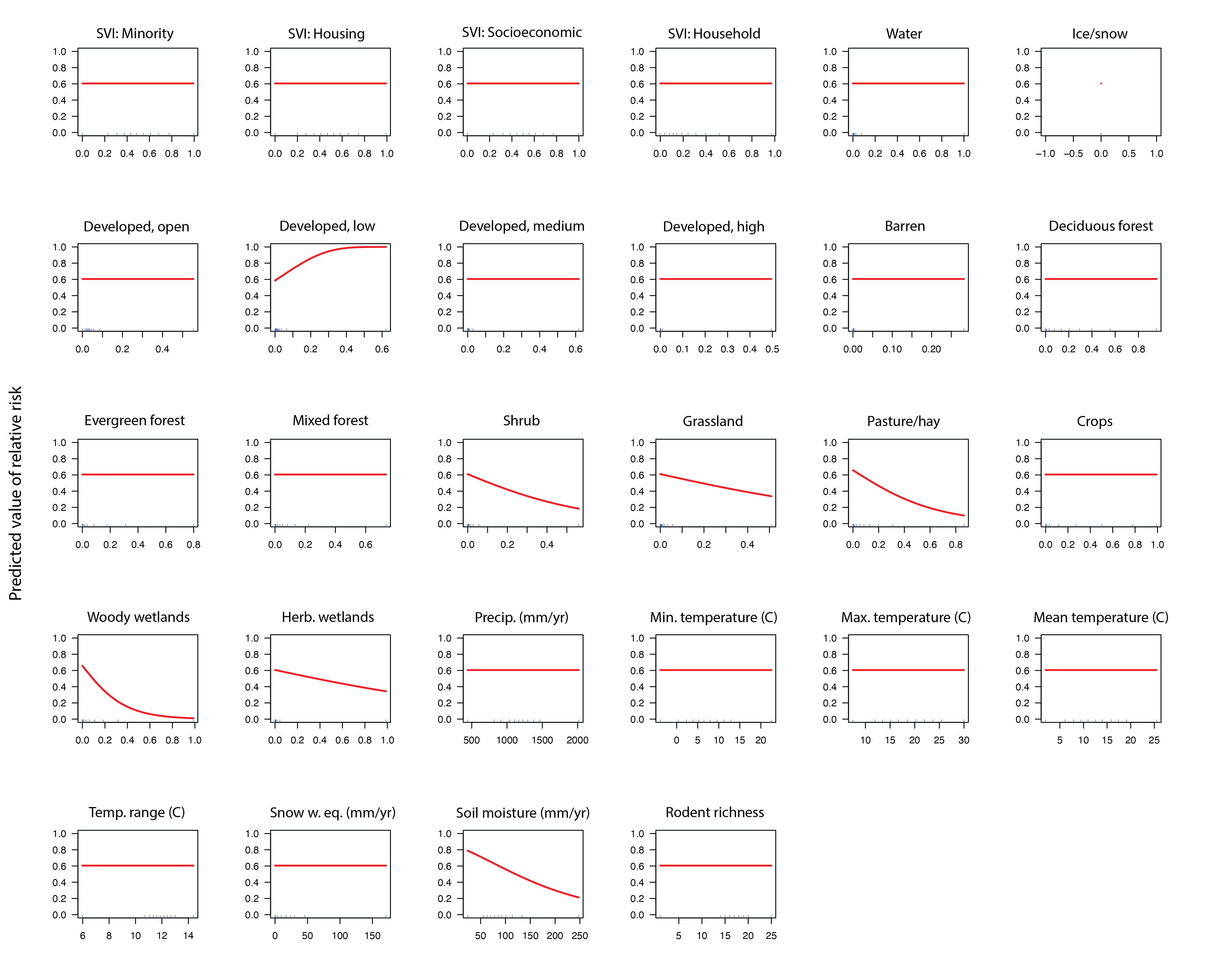
**

*Note:* SVI is Social Vulnerability Index; herb. is herbaceous; precip. is precipitation; min. is minimum; temp. is temperature; max. is maximum; w. is water; eq. is equivalent; m. is moisture.

**Figure S10.** Response curves for the social hantavirus risk model. Social Vulnerability Indices are 0-1.

**
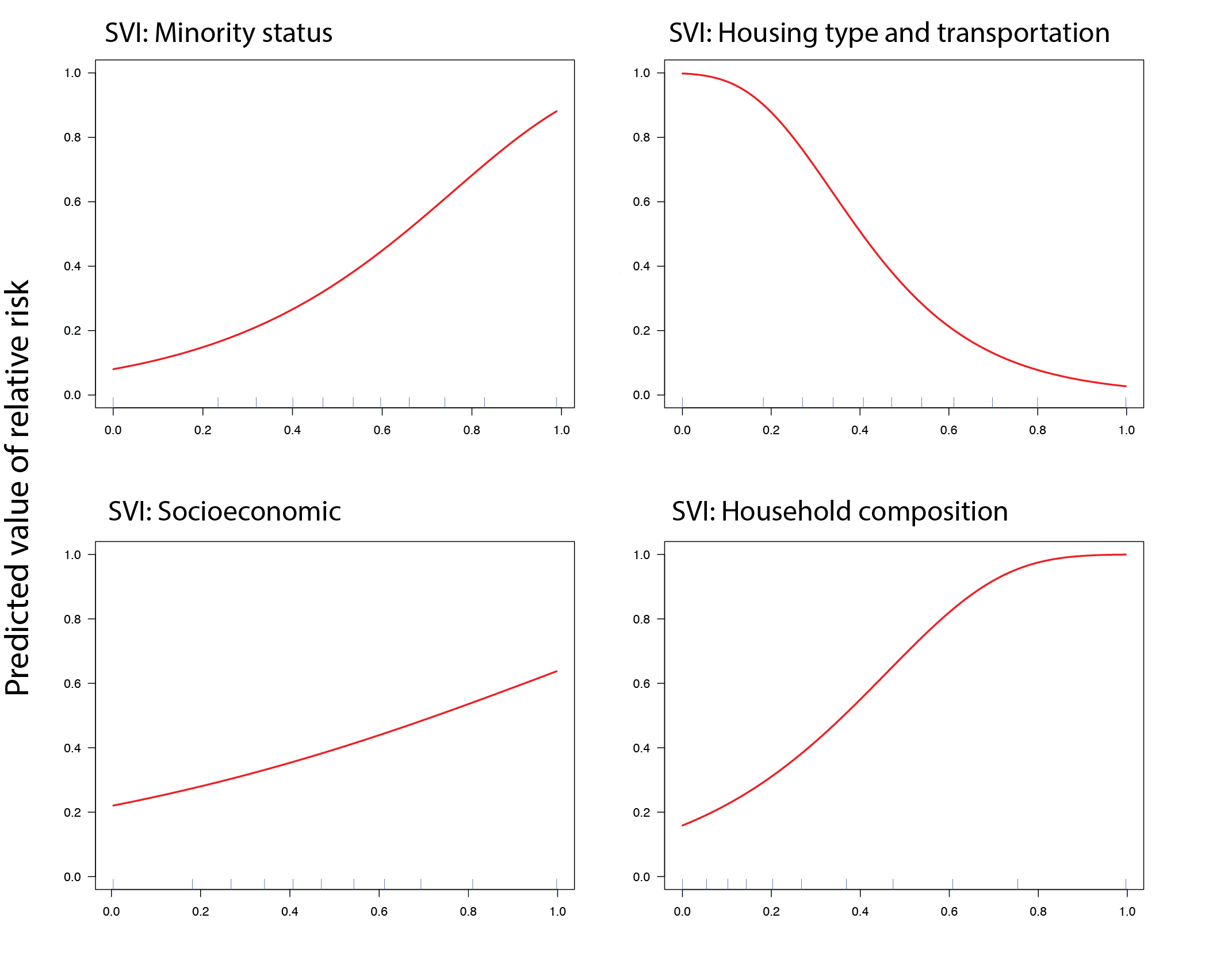
**

*Note:* SVI is Social Vulnerability Index.

**Figure S11.** Response curves for the land feature hantavirus risk model. Land use/cover variables are fractional values from 0-1. Rodent richness is on a unique scale.

**
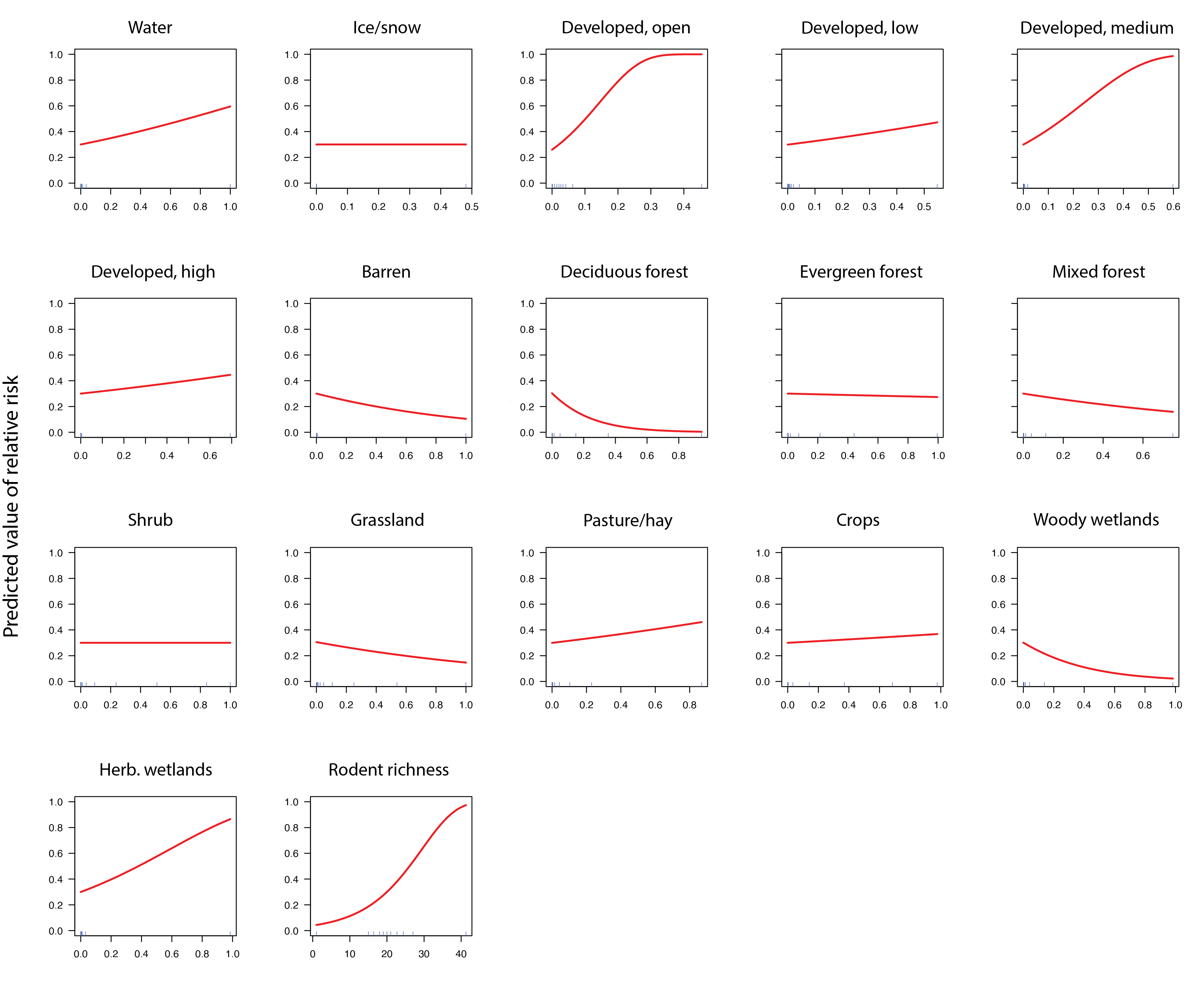

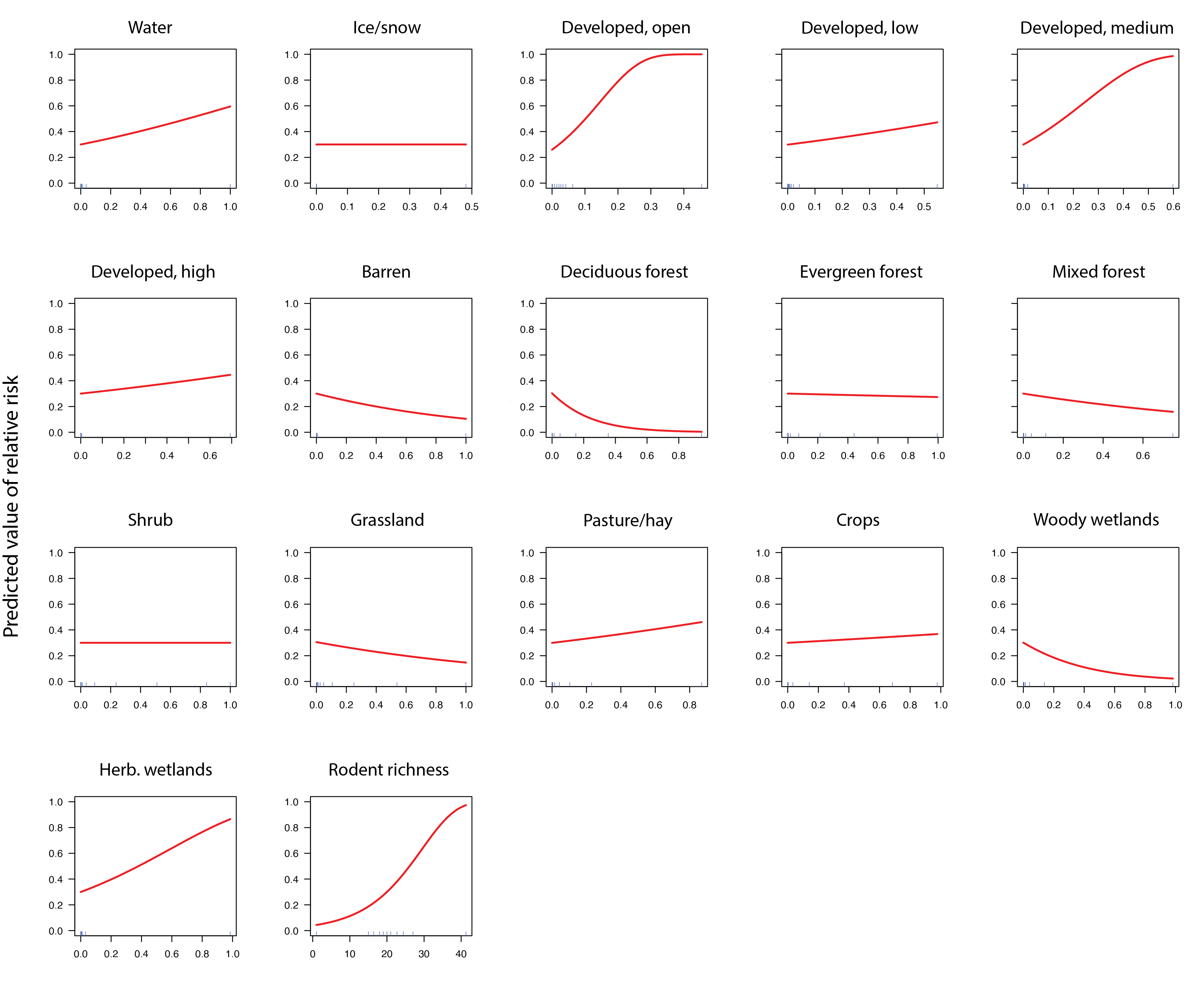

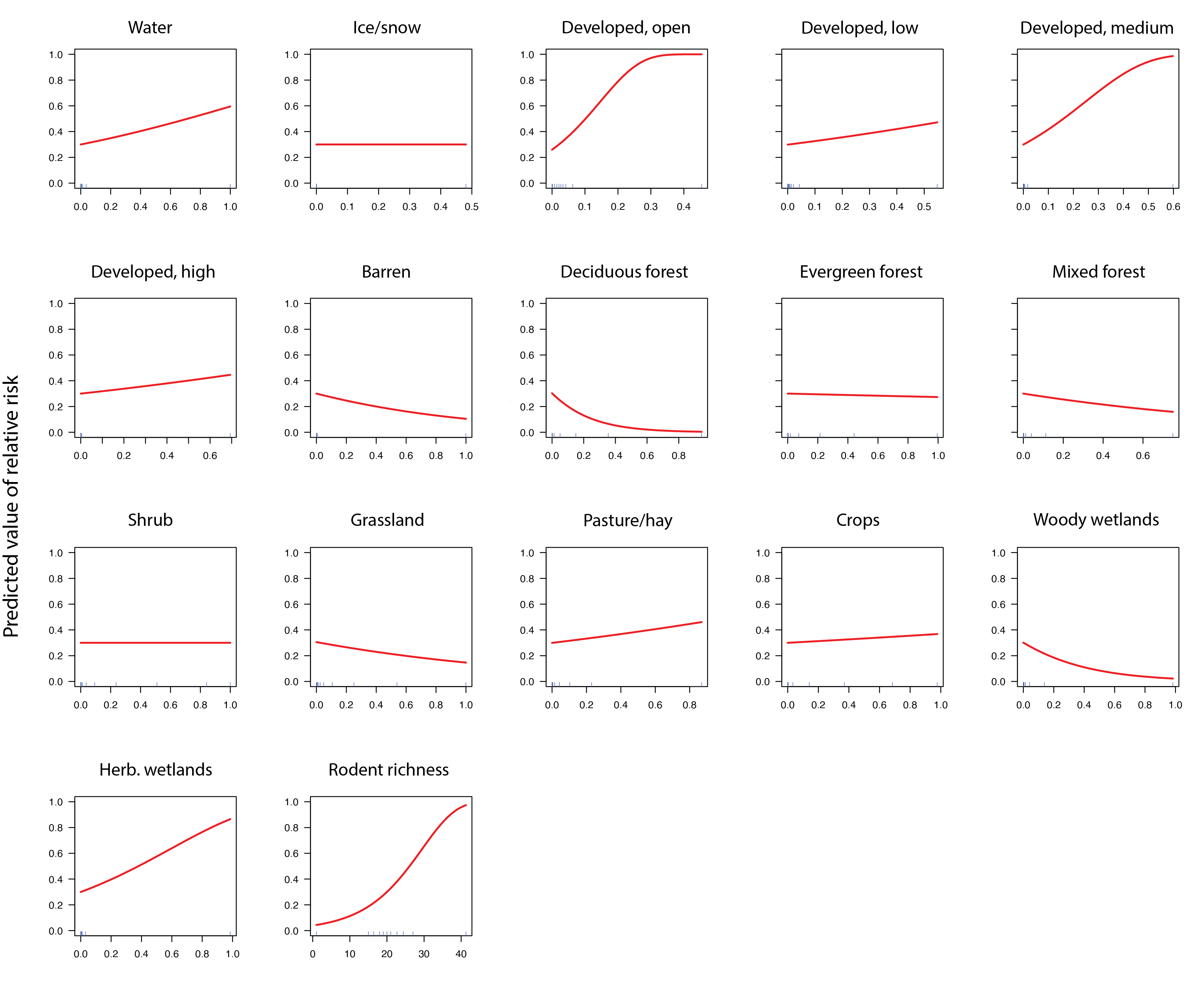

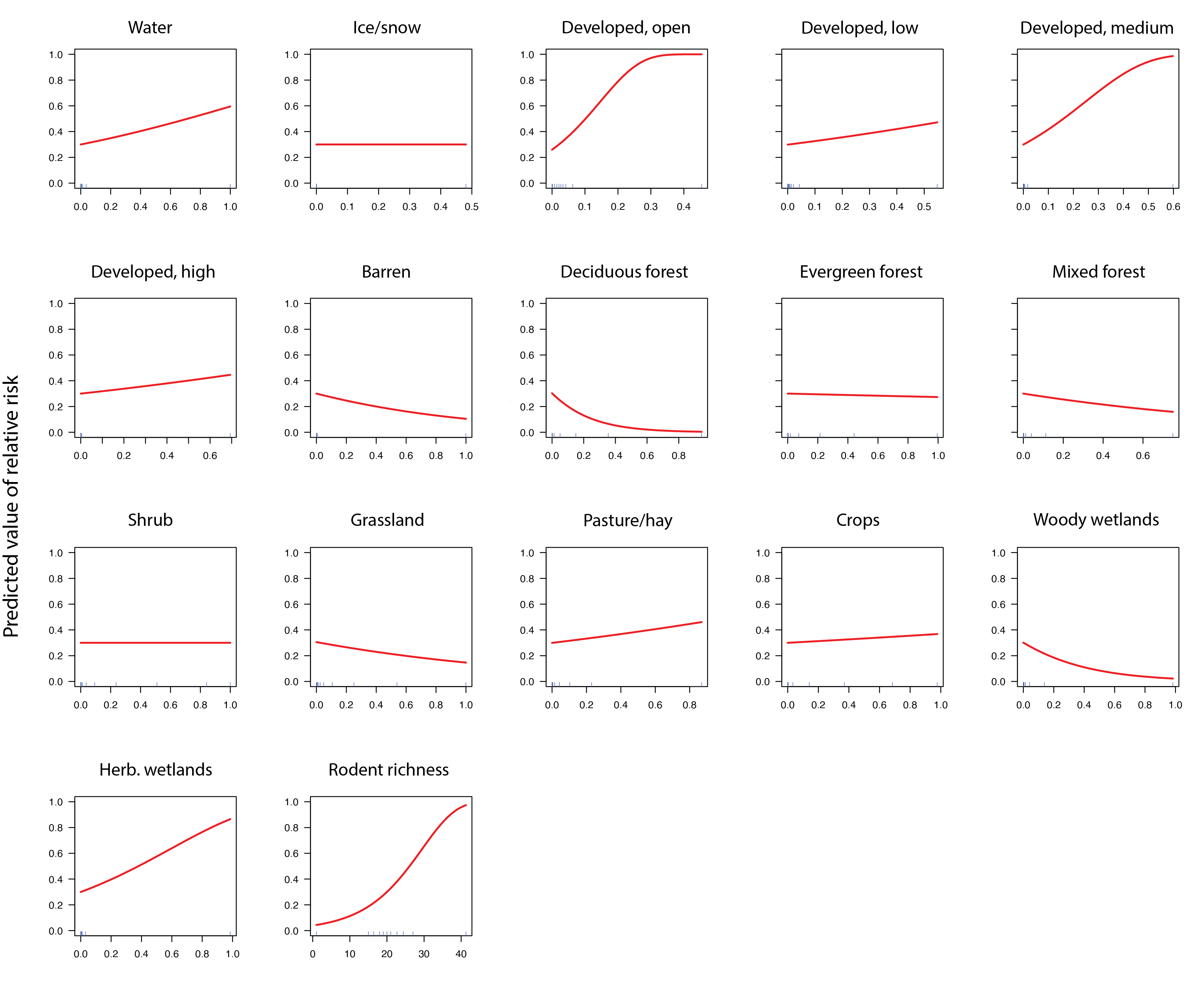

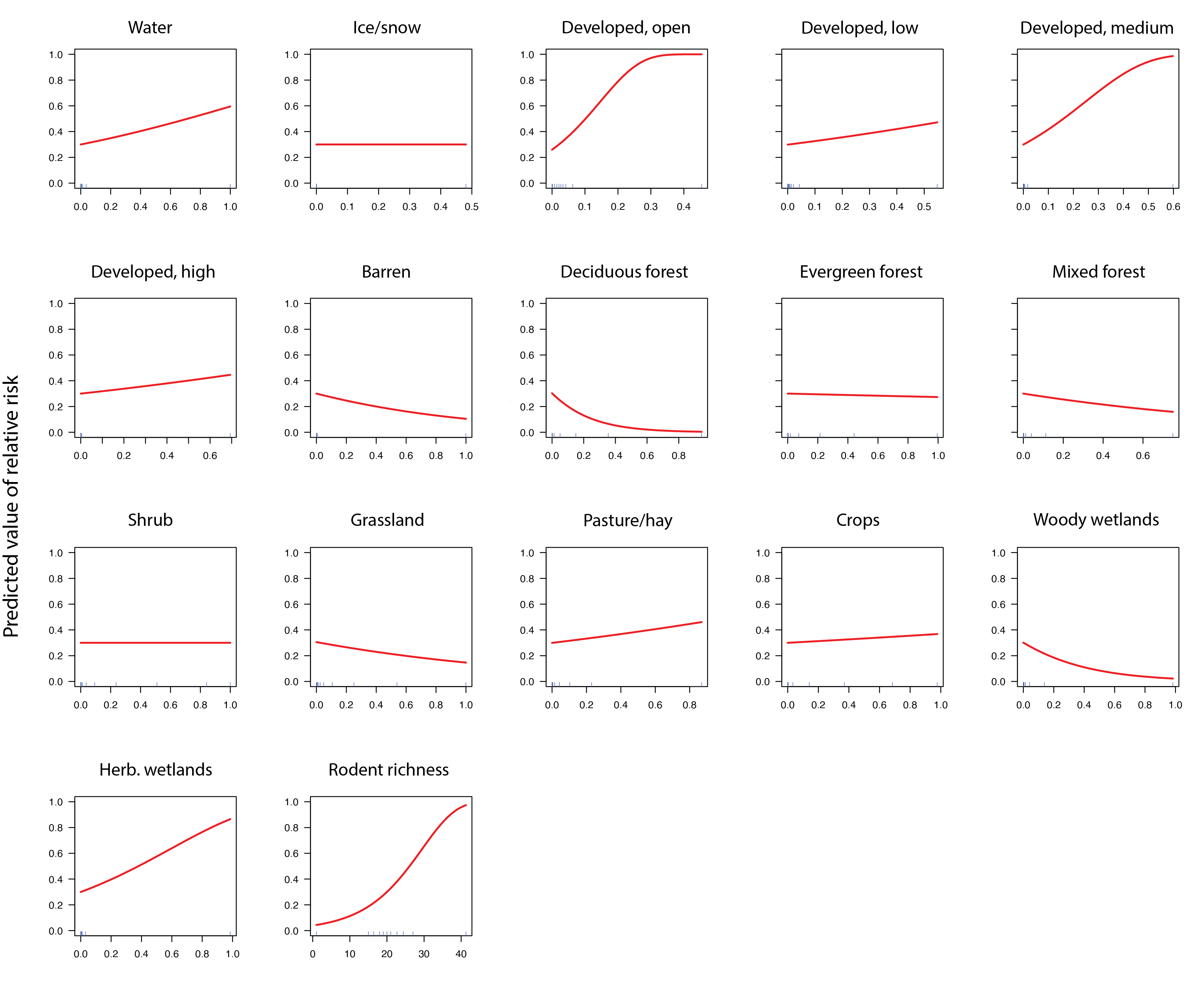
**

*Note:* Herb. is herbaceous.

**Figure S12.** Response curves for the climate hantavirus risk model. Each variable is on a scale unique to that variable.

**
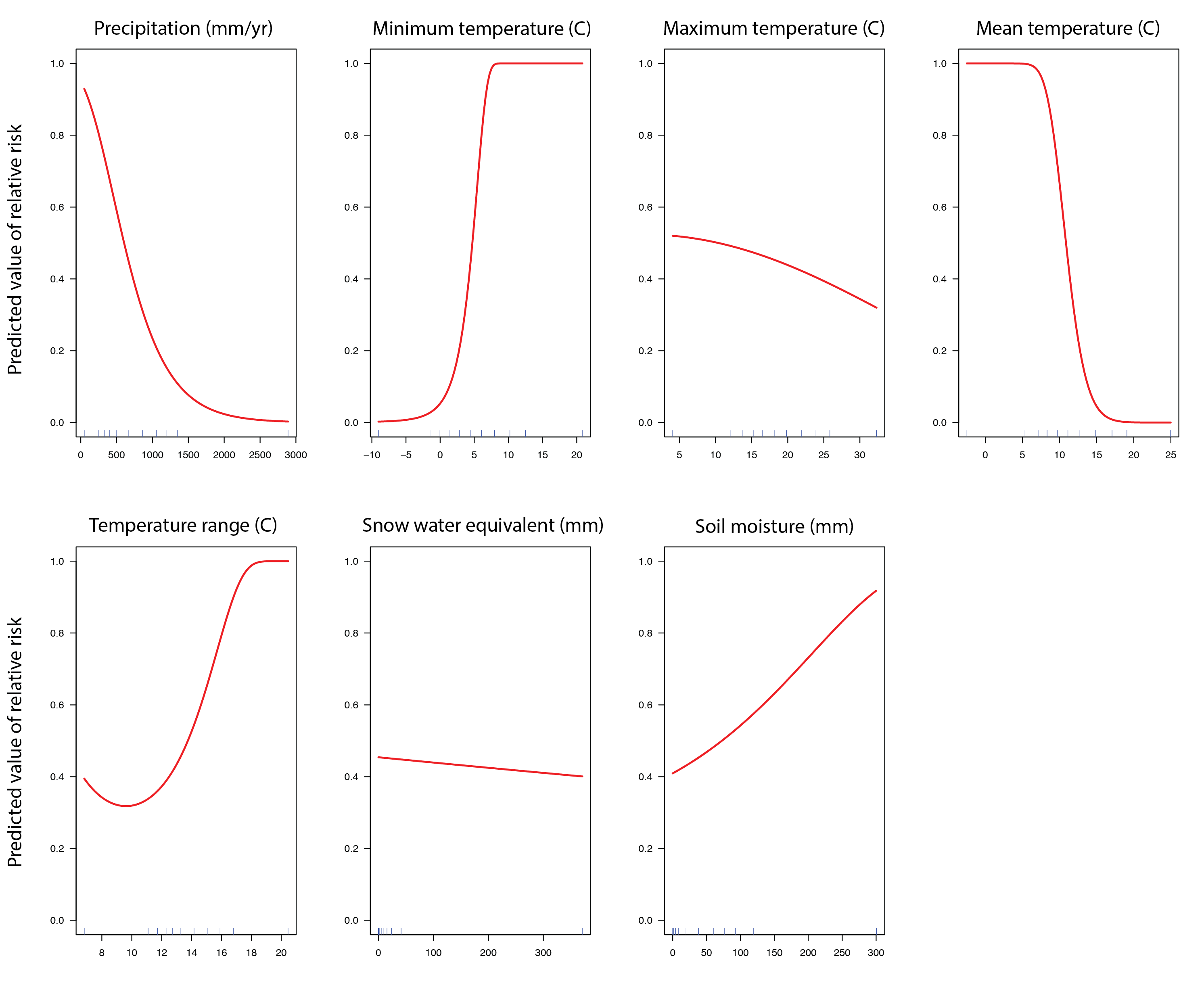
**

**Figure S13.** Maps of the range [0-1] between the maximum and minimum all-variable hantavirus risk amongst the ten bootstrapped replicates to show areas of high or low uncertainty for a) the contiguous U.S., b) the western U.S., and c) the eastern U.S. We label the range as such: 0.00 is “low uncertainty”, 0.25 is “moderate uncertainty”, and 0.50 is “high uncertainty.”


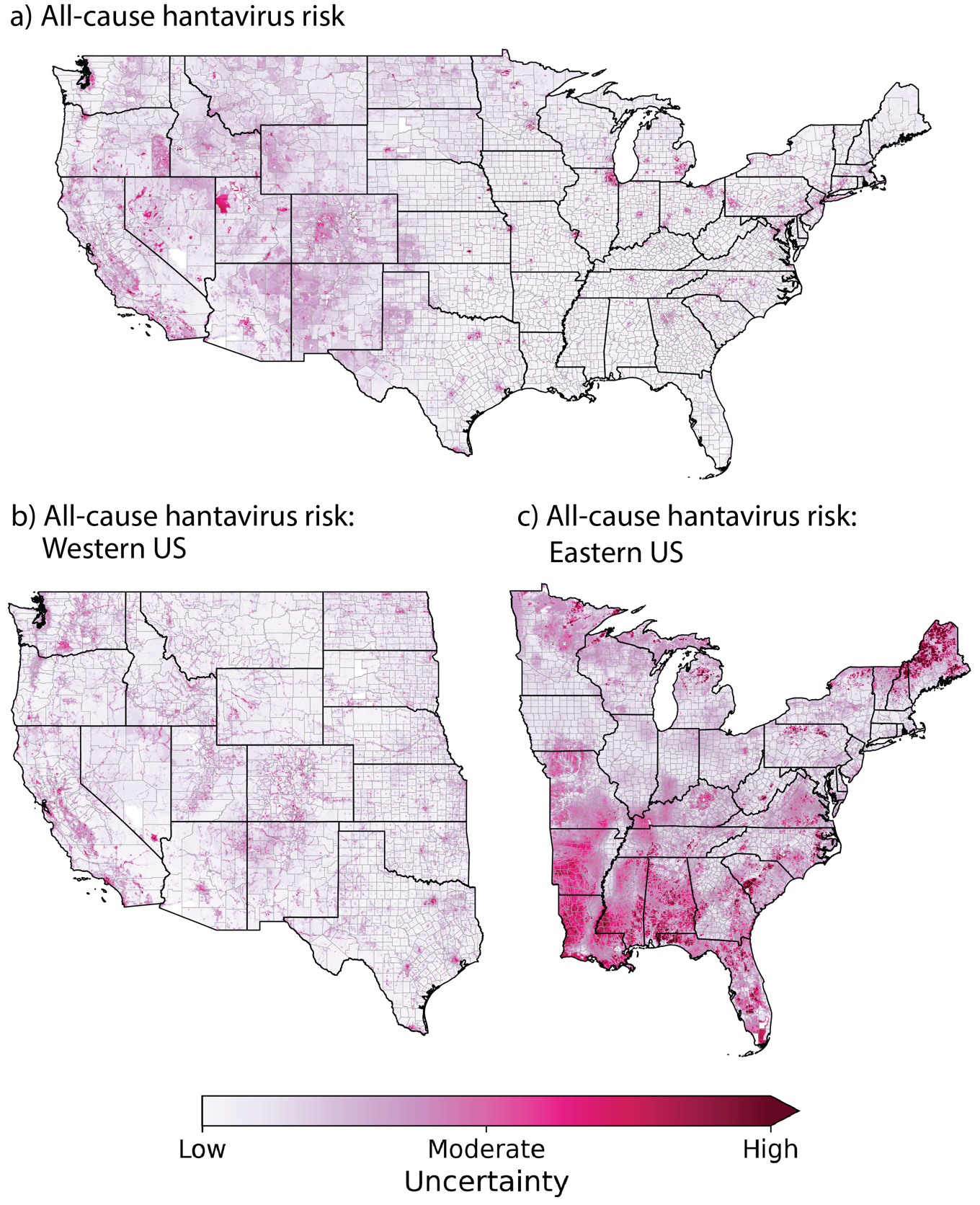


**Figure S14.** Maps of the range [0-1] between the maximum and minimum hantavirus risk models amongst the ten bootstrapped replicates to show areas of high or low uncertainty for the a) social risk model, b) land feature risk model, and c) climate risk model. We label the range as such: 0.00 is “low uncertainty”, 0.25 is “moderate uncertainty”, and 0.50 is “high uncertainty.”


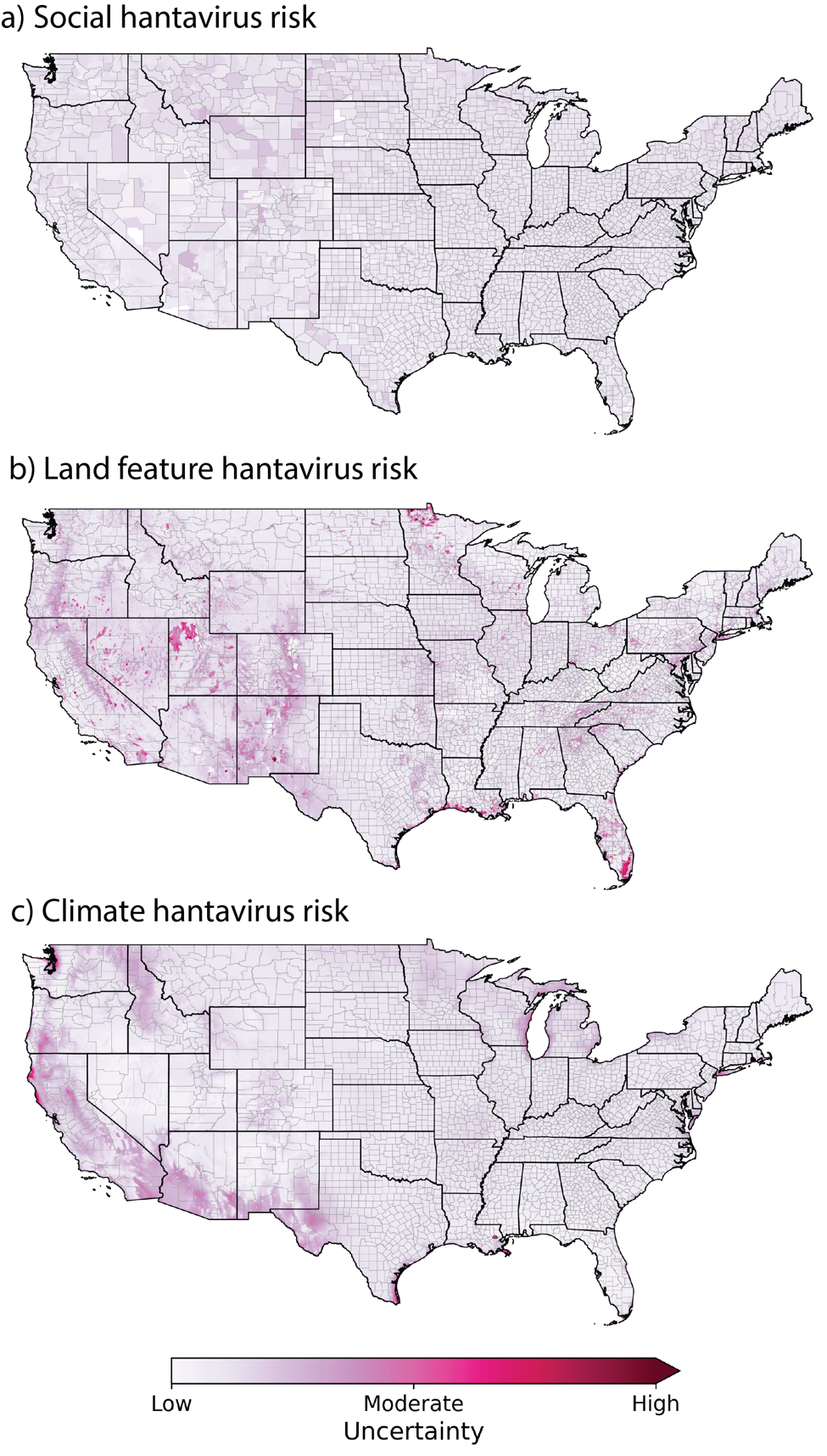

Supplement: Supporting Information — An additional Supporting Information file contains Tables S1–S2 and Figures S1–S14. Table S1. Final specifications and performance metrics for each risk model. Table S2. Mean percent variable contribution of each environmental variable averaged across the ten10 bootstraps of the top model of hantavirus risk. Figure S1. Hantavirus case reports before filtering. There were 575 cases from 1993 to 2022 that had latitude and longitude identified at the town-level. Figure S2. Four social risk variables included in the all-variable and social risk models from the U.S. Social Vulnerability Index Grids. Index values range from 0 to 1 based on their percentile position among all census tracts in the US, where 1 indicates the highest vulnerability. The four subsets of vulnerability are: (a) socioeconomic, (b) household composition, (c) minority status, and (d) housing type and transportation. Figure S3. First set of eight of the sixteen16 fractional land cover and land use variables (range 0–1) from the NLCD included in the all-variable and land feature risk models, including (a) water, (b) perennial ice/snow, (c) developed, open space, (d) developed, low intensity, (e) developed, medium intensity, (f) developed, high intensity, (g) barren land, and (h) deciduous forest. Figure S4. Second set of eight of the sixteen16 fractional land cover and land use variables (range 0–1) from the NLCD included in the all-variable and land feature risk models, including (a) evergreen forest, (b) mixed forest, (c) shrub/scrub, (d) grassland/herbaceous, (e) pasture/hay, (f) cultivated crops, (g) woody wetlands, and (h) emergent herbaceous wetlands. Figure S5. Rodent richness included in the all-variable and land feature risk models. Figure S6. Seven climate risk variables included in the all-variable and (a) precipitation, (b) minimum temperature, (c) maximum temperature, (d) mean temperature, (e) temperature range, (f) snow water equivalent, and (g) soil moisture. Figure S7. Response curves f [file 7126411.f1.docx]
